# ﻿The Zn(II)_2_-Cys_6_-type zinc finger protein AoKap7 is involved in the growth, oxidative stress and kojic acid synthesis in *Aspergillus oryzae*

**DOI:** 10.3897/imafungus.16.153994

**Published:** 2025-09-25

**Authors:** Ting Qiu, Ziming Chen, Huanxin Zhang, Bangfu Deng, Lihua Yao, Zhe Zhang

**Affiliations:** 1 College of Life Sciences, Jiangxi Key Laboratory of Natural Microbial Medicine Research, Jiangxi Science and Technology Normal University, Nanchang, 330013, China Jiangxi Science and Technology Normal University Nanchang China; 2 Institute of Horticulture, Jiangxi Academy of Agricultural Sciences, Nanchang, 330200, China Jiangxi Academy of Agricultural Sciences Nanchang China

**Keywords:** *Aspergillus oryzae*, CRISPR in *A. oryzae*, gene cluster, kojic acid, secondary metabolite

## Abstract

Although the kojic acid gene cluster in *Aspergillus oryzae* was identified in 2010, the functions of neighbouring genes remain poorly understood. Here, we characterise *Aokap7*, a gene adjacent to this cluster that encodes a novel nucleus-localised zinc finger protein without transcriptional activation activity. Disruption of *Aokap7* markedly accelerated spore germination, hyphal growth, and conidial formation, but impaired kojic acid production. Overexpression of *kojR* or *laeA* restored kojic acid production in the *Aokap7* disruption strain, whereas their disruption abolished it, indicating that *Aokap7* regulates kojic acid production by *kojR* and *laeA*. Furthermore, disrupting *Aokap7* in the *AozfA* overexpression or disruption strains reversed their kojic aicd production, suggesting that *Aokap7* acts downstream of *AozfA*. The *Aokap7* mutant also exhibited reduced expression of reactive oxygen species (ROS)-scavenging genes and heightened oxidative stress sensitivity. Biochemical assays revealed that *Aokap7* preferentially binds to the motif 5’-CGGCTCGG-3’, and directly interacts with the *AoGPX1* promoter. Disruption of *AoGPX1* increased the sensitivity of *A. oryzae* to oxidative stress. Our findings elucidate the pivotal role of *Aokap7* in coordinating growth, oxidative stress response and kojic acid production, advancing understanding of the regulatory network of kojic acid synthesis in *A. oryzae*.

## ﻿Introduction

Kojic acid, a valuable secondary metabolite originating from *Aspergillus oryzae* mycelium, showcases a wide range of applications spanning cosmetics, food, medicine, agriculture and various industries (Maruyama and Kitamoto 2011; Park et al. 2019; Chib et al. 2023; Felipe et al. 2023; Sharma et al. 2023). Currently, the predominant method for kojic acid production involves aerobic fermentation with *A. oryzae*. Given the extensive range of applications and the rapid expansion of the kojic acid market (Chib et al. 2023), there is a pressing need to elucidate the regulatory mechanisms governing kojic acid synthesis in *A. oryzae* to enhance its production.

Numerous genes involved in the synthesis and regulation of kojic acid have been characterised in *A. oryzae* (Chib et al. 2023; Sharma et al. 2023). The pioneering identification of the kojic acid gene cluster in 2010 uncovered its core composition, comprising three principal genes: *kojA*, *kojR* and *kojT* (Terabayashi et al. 2010). KojA, a FAD-dependent oxidoreductase, has been proposed as a direct participant in kojic acid synthesis (Terabayashi et al. 2010). The transportation of produced kojic acid is facilitated by the major facilitator superfamily (MFS) transporter KojT (Terabayashi et al. 2010). Positioned between *kojA* and *kojT* within the kojic acid gene cluster, *kojR* encodes a Zn(II)_2_Cys_6_ zinc finger protein that regulates kojic acid synthesis by activating *kojA* and *kojT* (Terabayashi et al. 2010; Marui et al. 2011). Recently, the C_2_H_2_-type zinc-finger protein AoZFA is found to regulate kojic acid production through *kojR* (Chen et al. 2023). The C_2_H_2_-type zinc-finger protein AoKap5 directly binds to the *kojT* promoter to regulate *kojT* expression (Li et al. 2023). The global regulator LaeA regulates kojic acid production through influencing *kojA*, *kojR* and *kojT* (Oda et al. 2011). Additionally, several other factors, including KpeA, HirA, AoZip2, AoGld3, AoKap1 and AoKap2, have been discovered to be involved in kojic acid synthesis in *A. oryzae* (Arakawa et al. 2019; Zhang et al. 2019; Fan et al. 2020; Kudo et al. 2021; Li et al. 2021a; Li et al. 2022a). Collectively, these findings emphasise the pivotal role of the kojic acid gene cluster within the regulatory network governing kojic acid synthesis.

The comparative genomic analysis of *A. fumigatus* and *A. nidulans* has shed light on the potential presence of a gene cluster in *A. oryzae* that spans from AO090113000132 to AO090113000145 (Terabayashi et al. 2010). Positioned adjacent to *kojT* (AO090113000138), *Aokap4* (AO090113000139) encodes a MFS transporter that is known to participate in the transport of kojic acid as upstream of *kojT* (Chen et al. 2022a). Another discovery is the reported AoKap6, encoding an unknown protein, regulates kojic acid synthesis acting upstream of *kojA* (Chen et al. 2022b). However, the functional roles of most genes within the putative cluster, except from *kojA*, *kojR*, *kojT*, *Aokap4*, and *Aokap6*, remain largely elusive.

The *Aokap7* gene (AO090124000095), adjacent to the kojic acid biosynthetic cluster in *A. oryzae*, encodes a Zn(II)_2_Cys_6_-type zinc finger protein of unknown function. Here, we resolve its role by profiling *Aokap7* expression, subcellular localisation and transactivation activity, generating *Aokap7* mutants via the CRISPR/Cas9 system, and assessing the effects on fungal growth and kojic acid production. Additionally, we explored the genetic regulatory relationships between *Aokap7* and *AozfA*/*LaeA*/*kojR*, aiming to unravel the underlying regulatory mechanism of *Aokap7* in kojic acid biosynthesis. We further identified the DNA-binding motif of *Aokap7* and its direct target gene. These investigations provide valuable insights into the functional roles of genes near the kojic acid gene cluster and shed light on the regulatory mechanisms governing kojic acid synthesis in *A. oryzae*.

## ﻿Materials and methods

### ﻿Strains, media and culture conditions

The *A. oryzae* 3.042 strain (CICC 40092) served as the wild type (WT) and the recipient strain in this work. The uridine/uracil auxotrophic strain (Δ*pyrG*) was constructed by the CRISPR/Cas9 system, based on *A. oryzae* 3.042 strain in our previous study (Fan et al. 2020). The Czapek-Dox (CD) medium (2% glucose, 0.2% NaNO_3_, 0.1% KH_2_PO_4_, 0.05% MgSO_4_, 0.05% KCl, 0.05% NaCl, 0.002% FeSO_4_) was used for phenotype observation. The DPY medium (dextrin-polypeptone-yeast extract: 2% glucose, 1% peptone, 0.5% yeast extract, 0.5% KH_2_PO_4_, 0.05% MgSO_4_) containing 0.03% H_2_O_2_ or 0.5 mM menadione sodium bisulphate (MSB) was used for oxidative stress. For other stress treatments, the CD medium was supplemented with 1.2/1.5 M NaCl, 120/150 μg/ml SDS. For heat stress, the fungal strains grown on the CD agar plates were incubated at 40/42 °C. The kojic acid medium (10% glucose, 0.1% K_2_HPO_4_, 0.05% KCl, 0.05% MgSO_4_, 0.1% yeast extract) was used for the assessment of kojic acid production. The cultures were incubated at 30 °C.

### ﻿Construction of *Aokap7* disruption strain

For disruption of the *Aokap7* gene in *A. oryzae*, a 20-bp protospacer sequence targeting the *Aokap7* gene was designed using the CRISPRdirect tool (https://crispr.dbcls.jp/). The designed protospacer sequence was incorporated into the *A. oryzae U6* promoter through amplification with primer sets PU6-F and PU6-*Aokap7*-R using the plasmid pPTRII-Cas9-Aokap6 as a template (Chen et al. 2022b), yielding the DNA fragment PU6-*Aokap7*. Subsequently, the sgRNA and *A. oryzae U6* terminator, conjoined with the protospacer sequence of *Aokap7*, were amplified from the pPTRII-Cas9-Aokap6 plasmid using primers TU6-*Aokap7*-F and TU6-R, generating the DNA fragment *Aokap7*-TU6. The fusion of the two amplified fragments (PU6-*Aokap7* and *Aokap7*-TU6) was achieved through overlap PCR with primers PU6-F and TU6-R, resulting in the assembly of the fragment PU6-*Aokap7*-TU6. Subsequent to this, the PU6-*Aokap7*-TU6 fragment was inserted into the SmaI-cut pPTRII-Cas9 vector harbouring the pyrithiamine resistance marker *ptrA* (Li et al. 2021b), forming the plasmid pPTRII-Cas9-*Aokap7*. The resultant plasmid pPTRII-Cas9-*Aokap7* was introduced into the *A. oryzae* 3.042 strain using the protoplast transformation method (Maruyama and Kitamoto 2011). Transformants were subsequently selected on CD agar medium supplemented with 0.1 μg/ml pyrithiamine and their genetic modifications were validated through Sanger sequencing.

### ﻿Construction of complemented strain

To re-introduce *Aokap7* into the *Aokap7*-disrupted mutant, the WT genomic DNA, containing the 1,768-bp upstream sequence, open reading frame (ORF) of *Aokap7* and 964-bp downstream region, was amplified using CpEX1-*Aokap7*-F/R primers. This DNA fragment was subsequently cloned into the SpeI/HindIII-digested pEX1 vector bearing a pyrG marker (Nguyen et al. 2016), producing the complemented vector CpEX1-*Aokap7*. To facilitate the integration of the CpEX1-*Aokap7* plasmid into the *Aokap7* disruptant, a double mutant of *pyrG* and *Aokap7* was established by introducing the previously constructed plasmid pPTRII-Cas9-pyrG into the *Aokap7*-disrupted mutant (Li et al. 2022b). The resulting complemented plasmid CpEX1-*Aokap7* was then introduced into the dual mutant of *pyrG* and *Aokap7* using the protoplast method. Verification of the introduced DNA fragment was performed through genomic PCR with the C-*Aokap7*-F and CpEX1-R primers, followed by sequencing validation.

### ﻿Overexpression of *kojR*/*laeA*/*AozfA* in the *Aokap7* disruption strain

To overexpress *kojR*/*laeA* in the *Aokap7* mutant, the *kojR*/*laeA* overexpression strains were firstly constructed. The open reading frames of *kojR*/*laeA* were amplified from the genomic DNA of *A. oryzae* 3.042 strain using primers detailed in Suppl. material 1: table S1. Subsequently, these amplified sequences were inserted into the AflII/BamHI-cut pEX2B vector (Nguyen et al. 2017), generating the plasmids pEX2B-kojR/laeA. The sequencing plasmids were introduced into uridine/uracil auxotrophic *A. oryzae* 3.042 through PEG-mediated protoplast transformation to establish the *kojR*/*laeA* overexpression strains. The *AozfA* overexpression strains had been constructed in our previous study (Chen et al. 2023). Then the plasmid pPTRII-Cas9-*Aokap7* was transformed into the *kojR*/*laeA*/*AozfA* overexpression strains. Colonies were screened on CD agar plates supplemented with 0.1 μg/ml pyrithiamine and verified by sequencing to obtain Δ*Aokap7*-OE-kojR/laeA/AozfA strains.

### ﻿Disruption of *Aokap7* in the *kojR*/*laeA*/*AozfA* disrupted strain

To construct the double mutants of *Aokap7* and *kojR*/*laeA*/*AozfA*, the constructed plasmid pPTRII-Cas9-*Aokap7* was introduced into the protoplasts of *kojR*/*laeA*/*AozfA* mutants. The *kojR* and *AozfA* disruptants, engineered in our previous study, bear a 65-bp insertion and a 1-bp insertion in their transcripts, respectively (Chen et al. 2022b; Chen et al. 2023). To disrupt *laeA*, the previously constructed plasmid pPTRII-Cas9-laeA was transformed into the *A. oryzae* 3.042 strain using the protoplast method (Li et al. 2022a). Subsequent screening of transformants was conducted on CD agar medium supplemented with pyrithiamine, followed by sequencing of target sites to obtain disruption strains.

### ﻿Morphological analysis

Conidia suspensions (10^8^ conidia per ml) from fungal strains were point inoculated on CD plates. After cultivation for three days at 30 °C, colony diameters were measured and conidia were collected from 6-mm cores of each plate for quantification using a haemocytometer. To assess the growth rate, a mixture of 10 μl of spore suspension (10^8^ conidia per ml) and 190 μl of potato dextrose broth (PDB) provided by HKM (HuanKai Microbial, GuangDong, China) was introduced into a 96-well plate and cultured at 30 °C (Qu et al. 2024). The growth dynamics were continuously monitored by a microplate reader. The experiment was performed in biological triplicates. To detect conidia germination, *A. oryzae* conidia were inoculated into 40 μl of PDB media at 30 °C. The morphology of germinated conidia was observed by ZOE™ Fluorescent Cell Imager (BIO-RAD, Hercules, CA, USA) at 2, 4, 6 and 8 h. To observe fungal morphology, 3 μl of fungal suspensions (10^8^ conidia per ml) was spotted between two sterile coverslips on the CD agar medium and cultivated at 30 °C for three days. The coverslips with mycelium and conidia were observed under a microscope.

### ﻿Expression analysis

The total RNA was extracted from the mycelia grounded by liquid nitrogen using a Fungal Total RNA Extraction Kit (Omega, Norcross, GA, USA) in accordance with the manufacturer’s protocol. The synthesis of first-strand cDNA involved reverse-transcription of 1 μg of total RNA utilising the Prime Script™ RT Reagent Kit with gDNA Eraser (TaKaRa, Dalian, China). Quantitative polymerase chain reaction (qPCR) was performed using a SYBR Green PCR Kit (TaKaRa, Dalian, China) and a CFX96 Real-Time PCR Detection System (Bio-Rad, Hercules, CA, USA). The histone H1 gene (AO090012000496) was utilised as an endogenous reference. The calculation of gene expression levels was performed using the 2^−ΔΔCt^ method (Livak and Schmittgen 2001).

### ﻿Subcellular localisation

The coding sequence (CDS) of *Aokap7* was amplified and fused into the pEX2B-GFP vector containing the *A. oryzae amyB* promoter, creating *Aokap7*-GFP fusion construct. The resulting plasmid was transformed into protoplasts of uridine/uracil auxotrophic *A. oryzae* 3.042. Transformants were confirmed by PCR and fluorescence signals. Spores of the fungal strain were spread on PDA agar medium and cultivated at 30 °C for 48 h. Mycelia were collected and washed with phosphate-buffered saline (PBS) and stained by 1 μg/ml 4,6-diamidino-2-phenylindole (DAPI) for 2 h. The fluorescence signals were analysed using a laser confocal scanning microscope.

### ﻿Transactivation activity assay of *Aokap7*

The CDS of *Aokap7* was cloned and inserted into the pGBKT7 vector (BD), generating the BD-*Aokap7* plasmid. For construction of BD-*Aokap7*-VP64 plasmid, the sequence of VP64 domain was cloned into the pGBKT7 vector, resulting in the BD-VP64 vector, and then the *Aokap7*CDS was inserted into the BD-VP64 vector, producing BD-*Aokap7*-VP64 plasmid. The resultant vectors were transformed into the AH109 yeast strain. After cultured on SD/-Trp dropout plates at 30 °C for 4 d, the transformed cells were transferred on SD/-Ade-His-Trp dropout plates to observe their growth.

### ﻿Kojic acid analysis

To assess the production of kojic acid, conidia suspensions (10^8^ conidia per ml) were inoculated in 40 ml of kojic acid liquid medium and cultivated for seven days with shaking at 200 rpm and 30 °C. Subsequently, the quantification of kojic acid content in the supernatant derived from fungal strains was conducted using the colorimetric method (Li et al. 2022a). The yield of kojic acid was quantified as g/l.

### ﻿Transcriptome analysis

The mycelia of WT and Δ*Aokap7*-1 strains were collected for transcriptome analysis after cultivation for three days in kojic acid liquid medium. Two biological replicates were performed. RNA extraction, construction of cDNA library, and next-generation sequencing were performed by Shanghai Majorbio Bio-pharm Technology Company (Shanghai, China) on an Illumina NovaSeq X Plus platform. The raw data were analysed using the customised Majorbio Cloud platform. Volcano plots and bubble plots were generated using the package ggplot2 version 3.5.1 and heatmaps were created by the package pheatmap version 1.0.12. Differential expression genes (DEGs) were identiﬁed using |log_2_(fold change)| ≥ 1 and Padjust < 0.05. Goatools (https://github.com/tanghaibao/GOatools) was used for GO analysis.

### ﻿Yeast one-hybrid assay

To identify the DNA motifs bound by *Aokap7*, we firstly constructed a random DNA insertion library, based on the plasmid pHIS2 using recombination in yeast as described previously (Jingwen et al. 2023). Briefly, the double strand insertion sequence was amplified by PCR reaction using a single DNA sequence containing 7 nt random sequence as a template with its complementary primer. The obtained double strand insertion sequence and SmaI-linearised pHIS2 vector were co-transformed into the yeast strain Y187 according to the manufacturer’s protocol of Yeastmaker™ Yeast Transformation System 2 (Clontech). Subsequently, the full-length CDS of *Aokap7* was cloned and ligated into the pGADT7 vector (AD), generating the AD-*Aokap7* plasmid. The recombined AD-*Aokap7* plasmid was transformed into the Y178 yeast strains containing the random DNA insertion prey library. The transformants were cultured on SD/-His-Leu-Trp dropout plates with 100 mM 3-aminotriazole (3-AT). The positive clones were used as templates to amplify random DNA insertion sequences using primers pHis2-F/R and then the PCR products were sequenced to identify the potential motifs bound by *Aokap7*.

To further confirm the potential sequence recognised by *Aokap7*, three tandem copies of identified binding sequences were cloned into pHIS2 vector. These constructed plasmids together with AD-*Aokap7* were co-transformed into the Y178 yeast strain to determine whether *Aokap7* could interact with these identified motifs.

### ﻿Electrophoretic mobility shift assay (EMSA)

The cDNA fragment of *Aokap7* encoding amino acids 49–91 that contains the zinc finger domain was amplified and inserted into pGEX-4T-1 vectors to obtain the glutathione S-transferase (GST) fusion protein (GST-*Aokap7*_zf_). Expression of GST and recombinant GST-*Aokap7*_zf_ proteins in the *Escherichia coli* strain BL21 (DE3) were induced using 1 mM isopropyl-β-D-1-thiogalactopyranoside for 16 h at 18 °C. GST and GST-*Aokap7*_zf_ proteins was puriﬁed using BeaverBeads GSH Kit (Beaver, Suzhou, China). Biotin-labelled and unlabelled probes were synthesised by General Biol Company (Anhui, China). The sequences are listed in Suppl. material 1: table S1. EMSA was performed according to the manufacturer’s instructions (Thermo Scientiﬁc, USA). Briefly, the mix containing purified proteins and the probes were incubated at room temperature for 20 min. Then the samples were run a 6.5% non-denaturing polyacrylamide gel in 0.5 X Tris-borate-EDTA buffer at 100 V for 60 min. Then the binding reactions were transferred to nylon membranes at 100 V for 45 min and cross-linked under a UV lamp for 20 min. The crosslinking membrane was blocked using blocking buffer for 20 min and then probed by streptavidin horseradish peroxidase conjugate. The signals were detected by a ﬂuorescence imaging system (Tanon, Shanghai, China).

### ﻿Creation of *AoGPX1* disrupted strain

To obtain the *AoGPX1* disrupted strain, the target sequence for the *AoGPX1* gene along with the *U6* promoter and *U6* terminator were inserted into the SmaI-cut pPTRII-Cas9 vector, resulting in the pPTRII-Cas9-AoGPX1 plasmid. The resulted plasmids were introduced into the *A. oryzae* 3.042 strain. Transformants were screened using pyrithiamine and confirmed by sequencing.

## ﻿Results

### ﻿Identiﬁcation and analysis of the *Aokap7* gene in *A. oryzae*

**Figure 1.**
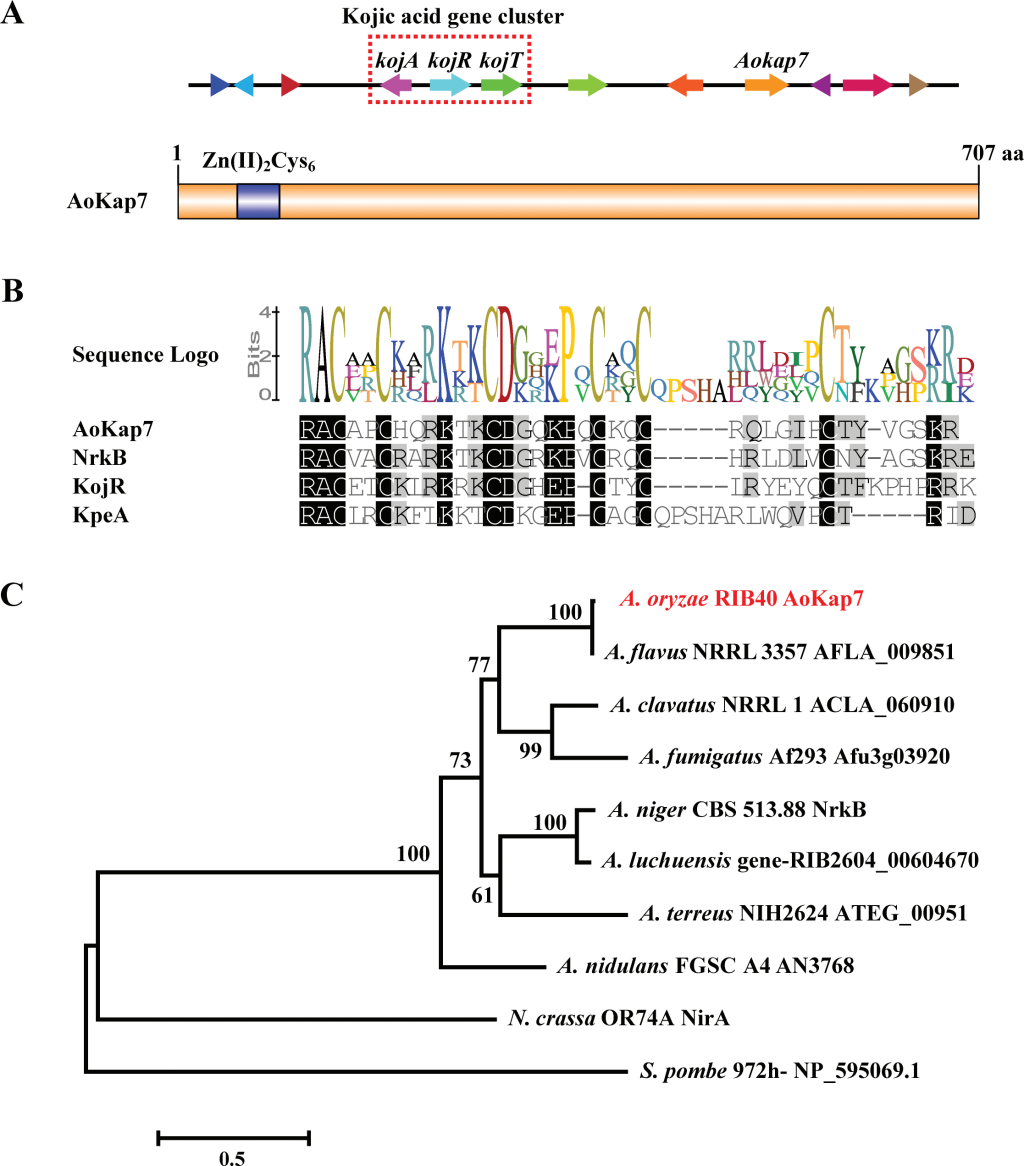
Bioinformatics analysis of *A. oryzae AoKap7*. **A** The location of *Aokap7* in kojic acid gene cluster in *A. oryzae* and a schematic diagram of the *AoKap7* protein. The Cys_2_His_2_ zinc-finger domain at the N-terminal end of the *AoKap7* protein is highlighted with a blue box and the numbers indicate the start and end sites of *AoKap7*; **B** A multiple sequence alignment of *AoKap7* and zinc finger proteins associated with kojic acid synthesis; **C** Phylogenetic tree analysing *AoKap7* homologues using MEGA7.0 with the neighbour-joining (NJ) method.

The *Aokap7* gene (AO090124000095), located adjacent to the kojic acid biosynthetic cluster, encodes a 707-amino acid protein (Fig. 1A). Utilising SMART and MOTIF tools, the domains within *Aokap7* were analysed, revealing the presence of a Zn(II)_2_-Cys_6_ zinc finger domain located at its Nterminal region (Fig. 1A). Multiple sequence alignment revealed a conserved zinc finger domain shared between *Aokap7* and the identified zinc finger proteins KojR, KpeA, and NrkB that were implicated in kojic acid synthesis (Fig. 1B). Phylogenetic analysis of the *Aokap7* protein together with its homologues revealed that these homologues could be divided into distinct subgroups, where *Aokap7* clustered closely with homologues from *A. flavus*, *A. clavatus*, and *A. fumigatus*, diverging from *A. niger*, *A. luchuensis* and *A. terreus* (Fig. 1C).

**Figure 2.**
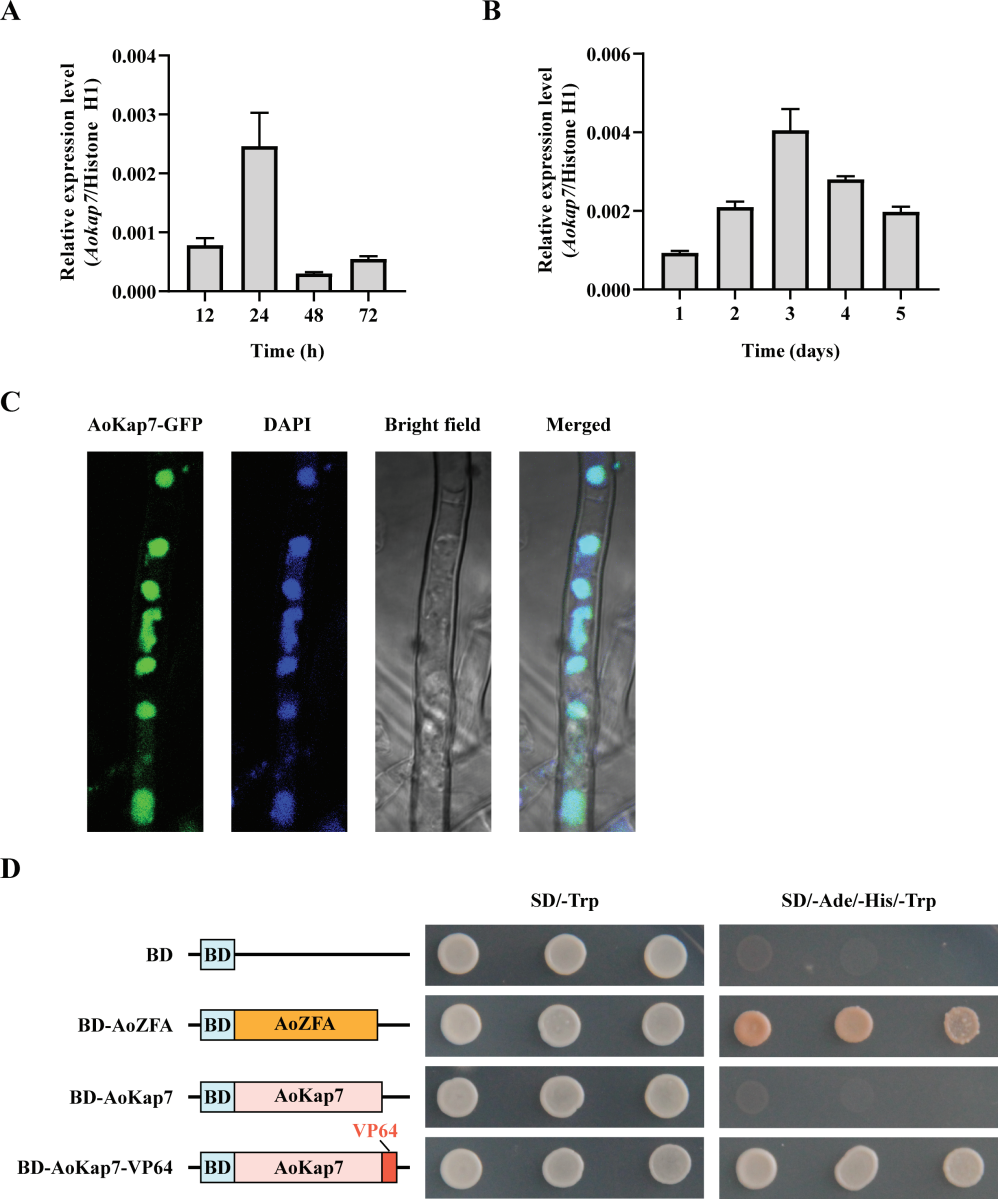
Characteristics of the *Aokap7* gene. **A** The mRNA levels of *Aokap7* in the wild-type cultured on the CD agar medium for indicated times; **B** Assessment of *Aokap7* expression in the wild-type strain cultured in the kojic acid liquid medium over several days; **C** Subcellular localisation of the *AoKap7* protein. GFP was fused to the C-terminal of *AoKap7* and nuclei were visualised using DAPI staining; **D** Transactivation activity assay of *AoKap7*. BD and BD fused with AoZFA served as negative and positive controls, respectively. The activation domain VP64 was fused with *AoKap7* to generate yeast bait construct BD-*Aokap7*-VP64.

To investigate the role of *Aokap7* in *A. oryzae*, an initial examination focused on the expression pattern of *Aokap7* in the wild type strain cultured in both solid and liquid media over specified durations. Reverse transcriptional quantitative PCR (RT-qPCR) analysis showed the transcript levels of *Aokap7* were markedly induced after 24 hours of cultivation on solid medium in contrast to the 12-hour mark, whereas the mRNA levels peaked at the third day of liquid medium culture (Fig. 2A, B). To delve deeper into the characteristics of *Aokap7*, we investigated its subcellular localisation by generating an *Aokap7*-GFP engineered strain. Fluorescing GFP signals of *Aokap7*-GFP fusion protein were observed in the nucleus of *A. oryzae* cells (Fig. 2C), consistent with the bioinformatics analysis that *Aokap7* is a Zn(II)_2_-Cys_6_ zinc finger protein predominantly functioning as a transcription factor. To further determine whether *Aokap7* had transcriptional activation activity, the full-length coding sequence of *Aokap7* was fused with the DNA-binding domain (BD) (Fig. 2D). The transformed yeasts harbouring BD-*Aokap7* failed to grow on SD/–Ade/–His/–Trp medium, suggesting the absence of transcriptional activity for *Aokap7*. Moreover, upon fusion with the VP64 domain (tetrameric repeats of the VP16 activation domain from *Herpes simplex virus*), *Aokap7* exhibited transcriptional activation activity (Fig. 2D).

### ﻿Disruption of the *Aokap7* gene affected the growth of *A. oryzae*

**Figure 3.**
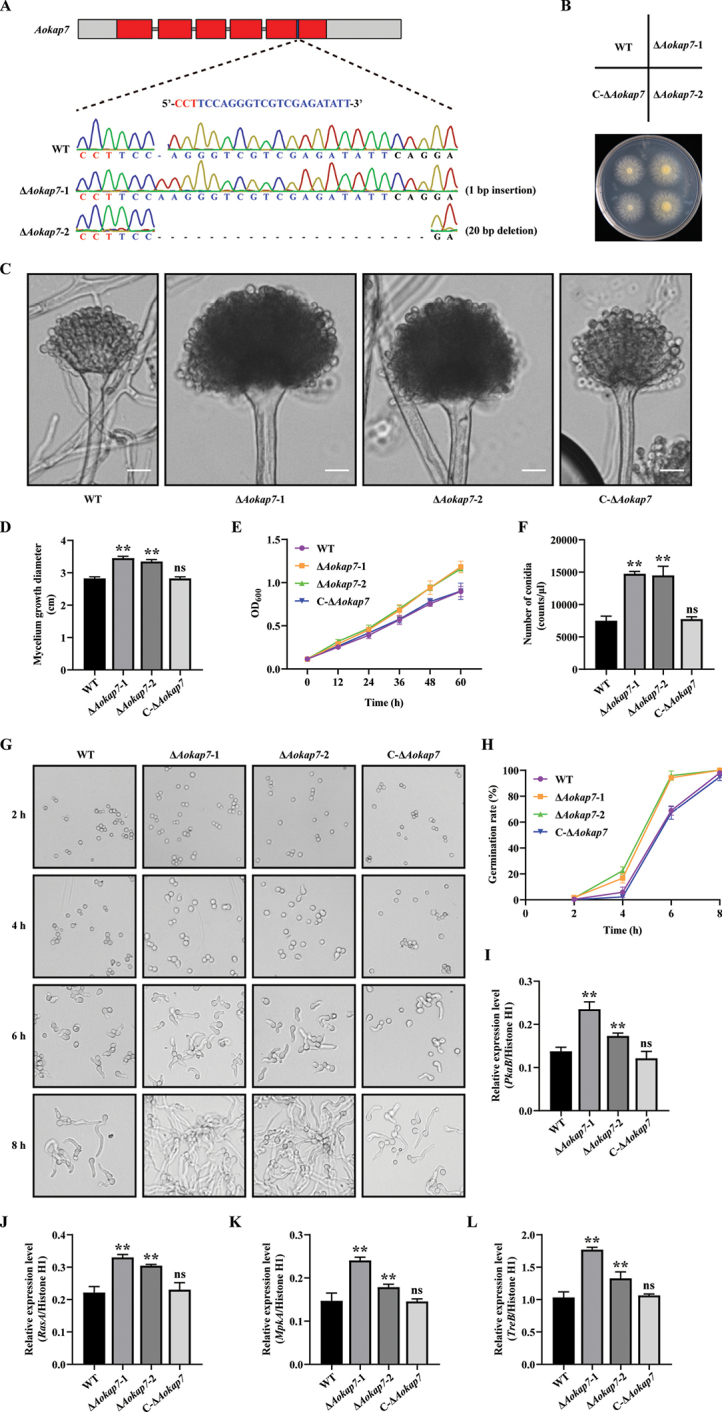
Impact of *Aokap7* disruption on mycelium growth, conidial production and spore germination. **A** Generation of *Aokap7* disruption strains using the CRISPR/Cas9 system. The Δ*Aokap7*-1 and Δ*Aokap7*-2 mutants exhibit a 1-bp insertion and a 20-bp deletion in the *Aokap7* gene, respectively. Red boxes denote the exons. The target sequence for *Aokap7* is shown in blue and its protospacer adjacent motif (PAM) is marked in red; **B** Growth profile of the wild-type strain (WT), *Aokap7* disruptants (Δ*Aokap7*-1 and Δ*Aokap7*-2) and *Aokap7* complement strain (C-Δ*Aokap7*) on CD agar medium at 30 °C for two days; **C** Conidiophores of the WT, Δ*Aokap7*-1, Δ*Aokap7*-2 and C-Δ*Aokap7* strains incubated on CD agar medium for 48 h; **D** Colonies diameters of the WT, Δ*Aokap7*-1, Δ*Aokap7*-2 and C-Δ*Aokap7* strains cultured on CD plates for two days; **(E)** The growth curve of the WT, Δ*Aokap7*-1, Δ*Aokap7*-2 and C-Δ*Aokap7* strains cultured in PDB at 30 °C; **(F)** Quantification of conidial production in fungal strains, including the WT, Δ*Aokap7*-1, Δ*Aokap7*-2 and C-Δ*Aokap7* strains; **(G)** The spore germination of the WT, Δ*Aokap7*-1, Δ*Aokap7*-2 and C-Δ*Aokap7* strains were visualised after cultivation in PDB for indicated times; **(H)** The conidial germination rate of the WT, Δ*Aokap7*-1, Δ*Aokap7*-2 and C-Δ*Aokap7* strains; **(I-L)** The transcriptional expression levels of spore germination-associated genes in the WT, Δ*Aokap7*-1, Δ*Aokap7*-2 and C-Δ*Aokap7* conidia cultured in PDB medium at 2 h. ** denotes statistically significant difference at *p* < 0.01 compared to the WT and “ns” indicates no significant difference. Scale bar: 10 μm.

To explore the biological function of *Aokap7* in *A. oryzae*, we utilised the CRISPR/Cas9 technology to disrupt the *Aokap7* gene. Following screening and verification, we successfully obtained two *Aokap7*-disrupted strains, namely Δ*Aokap7*-1 and Δ*Aokap7*-2 (Fig. 3A, B). The Δ*Aokap7*-1 and *Aokap7*-2 strains exhibited a 1-bp insertion and a 20-bp deletion in their transcripts, respectively, resulting in translational termination (Fig. 3A). Additionally, we generated a complemented strain, denoted as C-Δ*Aokap7*, by re-integrating the *Aokap7* gene into the Δ*Aokap7*-1 strain (Suppl. material 1: fig. S1). To elucidate the role of *Aokap7* in the growth of *A. oryzae*, the WT, Δ*Aokap7*-1, Δ*Aokap7*-2 and C-Δ*Aokap7* strains were cultivated on CD agar medium at 30 °C (Fig. 3B). After a two-day incubation, we observed that the *Aokap7* disruption strains formed larger conidial heads compared to the WT and C-Δ*Aokap7* strains, with the stalks of the *Aokap7* disruptants being thicker than those of both the WT and complemented strains (Fig. 3C). On the third day of cultivation, the colony diameters and conidia numbers were detected. As shown in Fig. 3D, the colony diameters of *Aokap7*-disrupted strains were larger compared with those of the WT and C-Δ*Aokap7* strains. Furthermore, upon inoculating spore suspensions into PDB, we observed that the growth curve of *Aokap7* disruptants expanded at a faster rate in contrast to that of the WT and C-Δ*Aokap7* strains (Fig. 3E). Consistent with morphological observations, the *Aokap7* disruptants displayed a significant increase in conidia numbers when compared to the WT strain (Fig. 3F). Additionally, we observed spore germination and found that the conidia of *Aokap7* disrupted strains germinated faster than the control WT and C-Δ*Aokap7* strains (Fig. 3G, H). The expression levels of spore germination-related genes, including *pkaB*, *rasA*, *mpkA* and *treB*, were significantly elevated in the *Aokap7* disruptants (Fig. 3I–L).

### ﻿Disruption of the *Aokap7* gene caused the declined production of kojic acid

**Figure 4.**
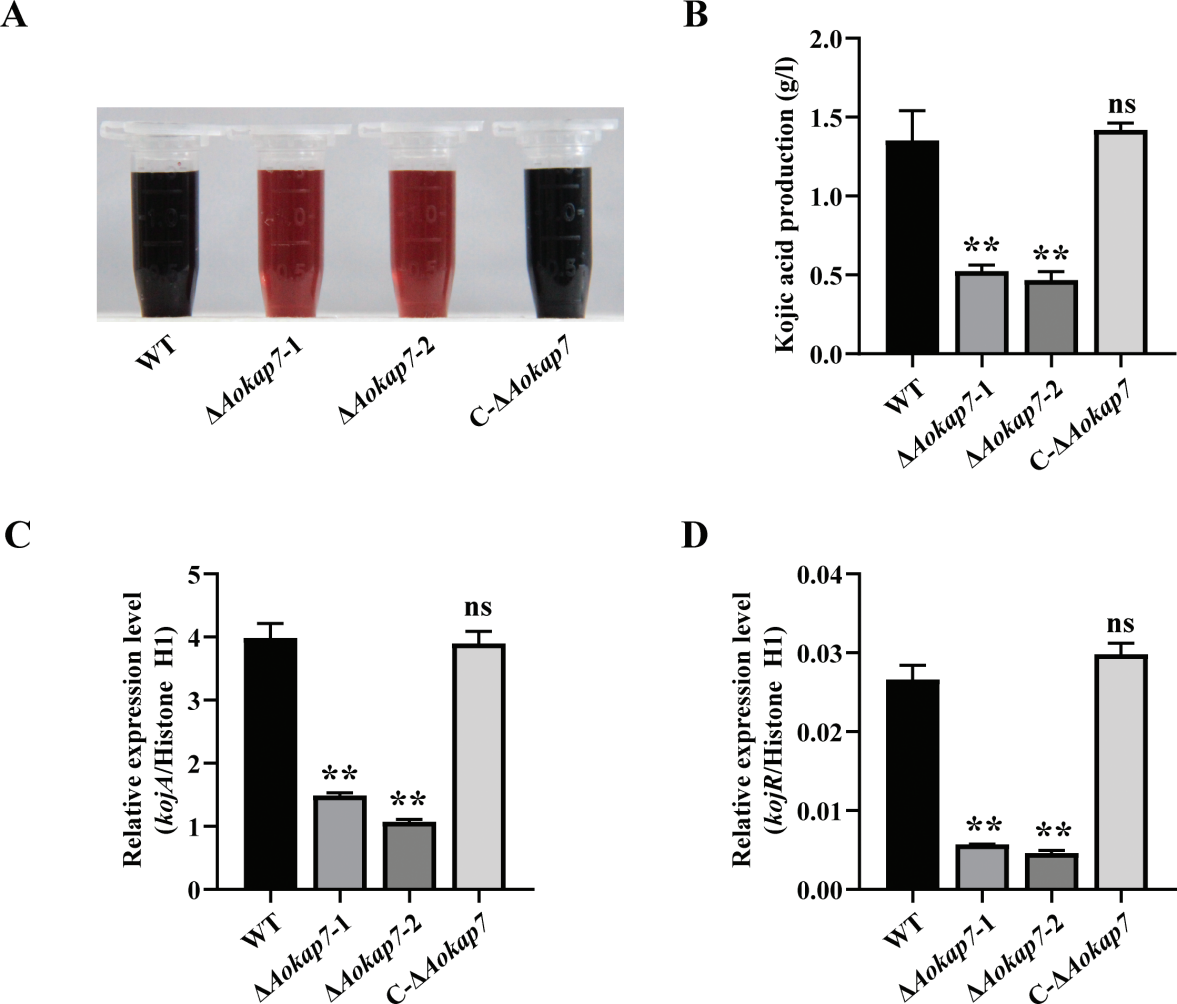
Impact of *Aokap7* disruption on kojic acid production. **A** Colorimetric analysis of kojic acid synthesis in the wild-type (WT), *Aokap7* disruptants (Δ*Aokap7*-1 and Δ*Aokap7*-2) and *Aokap7* complement strain (C-Δ*Aokap7*) following a seven-day incubation in kojic acid liquid medium at 30 °C. Kojic acid interaction with Fe^3+^ results in a red colouration; **B** Quantification of kojic acid production in the WT, Δ*Aokap7*-1, Δ*Aokap7*-2 and C-Δ*Aokap7* after fermentation for seven days; **C, D** The transcript levels of *kojA* (C) and *kojR* (D) in the WT, Δ*Aokap7*-1, Δ*Aokap7*-2 and C-Δ*Aokap7* cultured in kojic acid liquid medium for four days. Asterisks indicate significant differences compared to the WT strain, based on Student’s t-test (**p < 0.01; ns, no significant difference).

To investigate the effects of *Aokap7* on kojic acid production in *A. oryzae*, the WT, *Aokap7* mutants, and the complemented strain C-Δ*Aokap7* were inoculated into kojic acid liquid medium, and the fermentation process was carried out for seven days with shaking at 200 rpm. The presence of kojic acid was visually indicated by a red colour resulting from the reaction between kojic acid and Fe^3+^. The result showed that the *Aokap7* disruption strains exhibited lower intensity of red colour compared to the WT strain (Fig. 4A), indicating that disruption of *Aokap7* impairs kojic acid production. To further evaluate the impact on yield, we quantified the kojic acid production by utilising a FeCl_3_-colorimetric method. The result showed that the yield of kojic acid decreased by 63% in the *Aokap7* disruptants compared to the control WT strain, while the complemented strain C-Δ*Aokap7* exhibited restored kojic acid production similar to that of the WT strain (Fig. 4A, B), indicating that the diminished production of kojic acid in the *Aokap7* disruption strains is conclusively attributed to *Aokap7*. To gain insights into the potential relationship between the reduced kojic acid production and the kojic acid gene cluster, the expression levels of the key genes *kojA* and *kojR* were analysed by RT-qPCR. Total RNA was extracted from the fungal mycelium cultivated in kojic acid liquid medium for four days. We found that the transcript levels of *kojA* and *kojR* in the *Aokap7* disruption strains were decreased by 68% and 81% compared to the WT strain, respectively (Fig. 4C, D). These results suggest that the involvement of *Aokap7* in kojic acid synthesis is associated with *kojA* and *kojR*.

### ﻿*Aokap7* acts upstream of *kojR*

**Figure 5.**
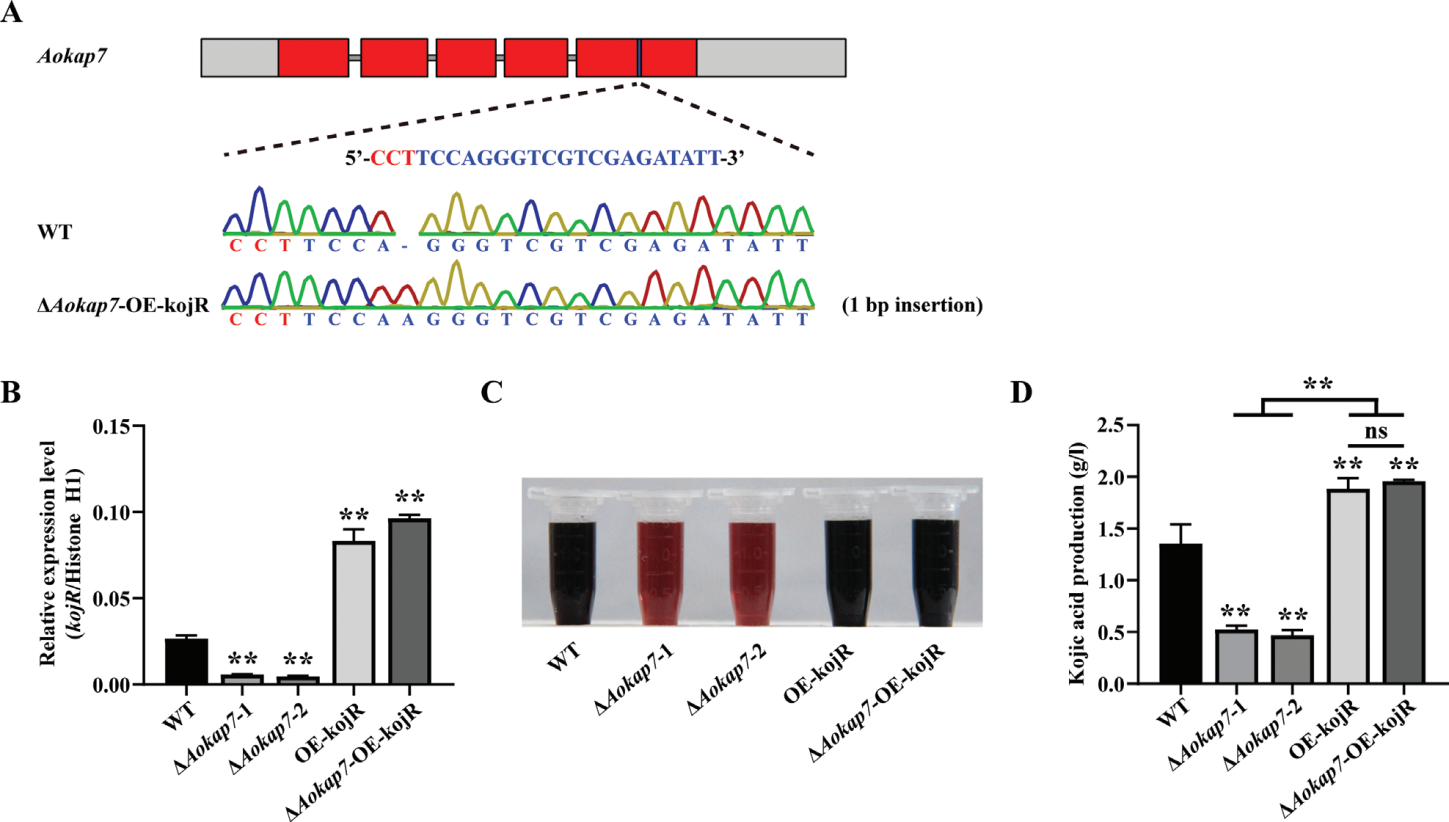
Restoration of kojic acid production through overexpression of *kojR* in the *Aokap7* disruption strain background. **A** A 1-bp insertion occurred in the *Aokap7* gene of the Δ*Aokap7*-OE-kojR strain, where *Aokap7* was disrupted and *kojR* was overexpressed. The target sequence for *Aokap7* and its protospacer adjacent motif (PAM) are indicated in blue and red, respectively; **B** Comparison of *kojR* transcript levels amongst the wild-type (WT), Δ*Aokap7*-1, Δ*Aokap7*-2, *kojR* overexpression strain (OE-kojR) and Δ*Aokap7*-OE-kojR cultured in kojic acid liquid medium for four days; **C** Colorimetric analysis of kojic acid production in the WT, Δ*Aokap7*-1, Δ*Aokap7*-2, OE-kojR and Δ*Aokap7*-OE-kojR strains following a seven-day incubation in kojic acid liquid medium; **D** Quantification of kojic acid yield from the WT, Δ*Aokap7*-1, Δ*Aokap7*-2, OE-kojR and Δ*Aokap7*-OE-kojR strains cultured in kojic acid liquid medium for seven days. Asterisks indicate statistically signiﬁcant differences compared to the WT strain, based on Student’s t-tests (**p < 0.01; ns, no significant difference).

**Figure 6.**
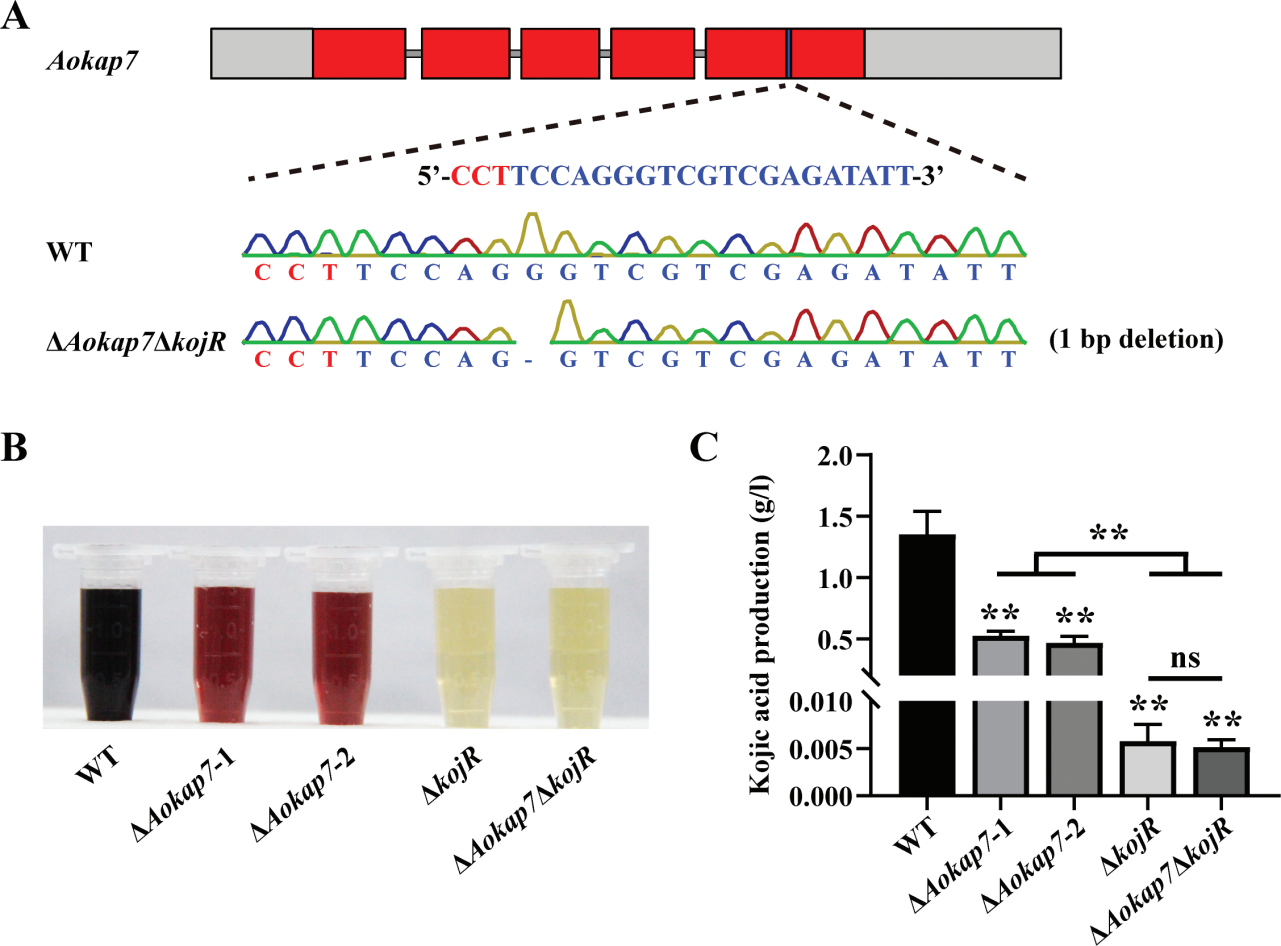
Effects of disruption of *kojR* in the *Aokap7* disruption strain on kojic acid production. **A** The double mutant of *Aokap7* and *kojR* was constructed, based on the *kojR* disruption mutant and it had a 1-bp deletion in the *Aokap7* transcript. The target sequence for *Aokap7* is highlighted in blue and its protospacer adjacent motif (PAM) is indicated in red; **B** Colour reaction of kojic acid produced by the wild-type (WT), Δ*Aokap7*-1, Δ*Aokap7*-2, Δ*kojR* and Δ*Aokap7*Δ*kojR* strains following a seven-day incubation in kojic acid liquid medium; **C** Quantitative analysis of kojic acid production in the WT, Δ*Aokap7*-1, Δ*Aokap7*-2, Δ*kojR* and Δ*Aokap7*Δ*kojR* strains after seven days of fermentation in kojic acid liquid medium. Asterisks indicate statistically signiﬁcant differences compared to the WT strain, based on Student’s t-tests (**p < 0.01; ns, no significant difference).

To elucidate the interaction between *Aokap7* and the genes within kojic acid gene cluster, we investigated the impact of *kojR* overexpression in the *Aokap7*-disrupted strain on kojic acid production, achieved through the generation of the Δ*Aokap7*-OE-kojR strain. The Δ*Aokap7*-OE-kojR strain contained a 1-bp insertion in the *Aokap7* transcript, where the expression level of *kojR* was increased by 3.1-fold (Fig. 5A, B). After seven days of fermentation, the Δ*Aokap7*-OE-kojR strain demonstrated comparable levels of kojic acid production to the OE-kojR strain (Fig. 5C, D), suggesting that overexpression of *kojR* can restore the diminished kojic acid production in the Δ*Aokap7* strain. To further validate the genetic interplay between *Aokap7* and *kojR*, a Δ*Aokap7*Δ*kojR* double disruptant was constructed, based on our previously constructed *kojR* disruption strain using the CRISPR/Cas9 system (Suppl. material 1: fig. S2). The Δ*Aokap7*Δ*kojR* strain harboured a 1-bp deletion in the *Aokap7* transcript, leading to premature translation termination (Fig. 6A). Subsequent evaluation of kojic acid production revealed that the Δ*Aokap7*Δ*kojR* strain displayed a Δ*kojR* strain-like yield of kojic acid (Fig. 6B, C). These results clearly indicate that *Aokap7* plays a role upstream of *kojR*.

### ﻿*Aokap7* positively regulated kojic acid production through *laeA*

**Figure 7.**
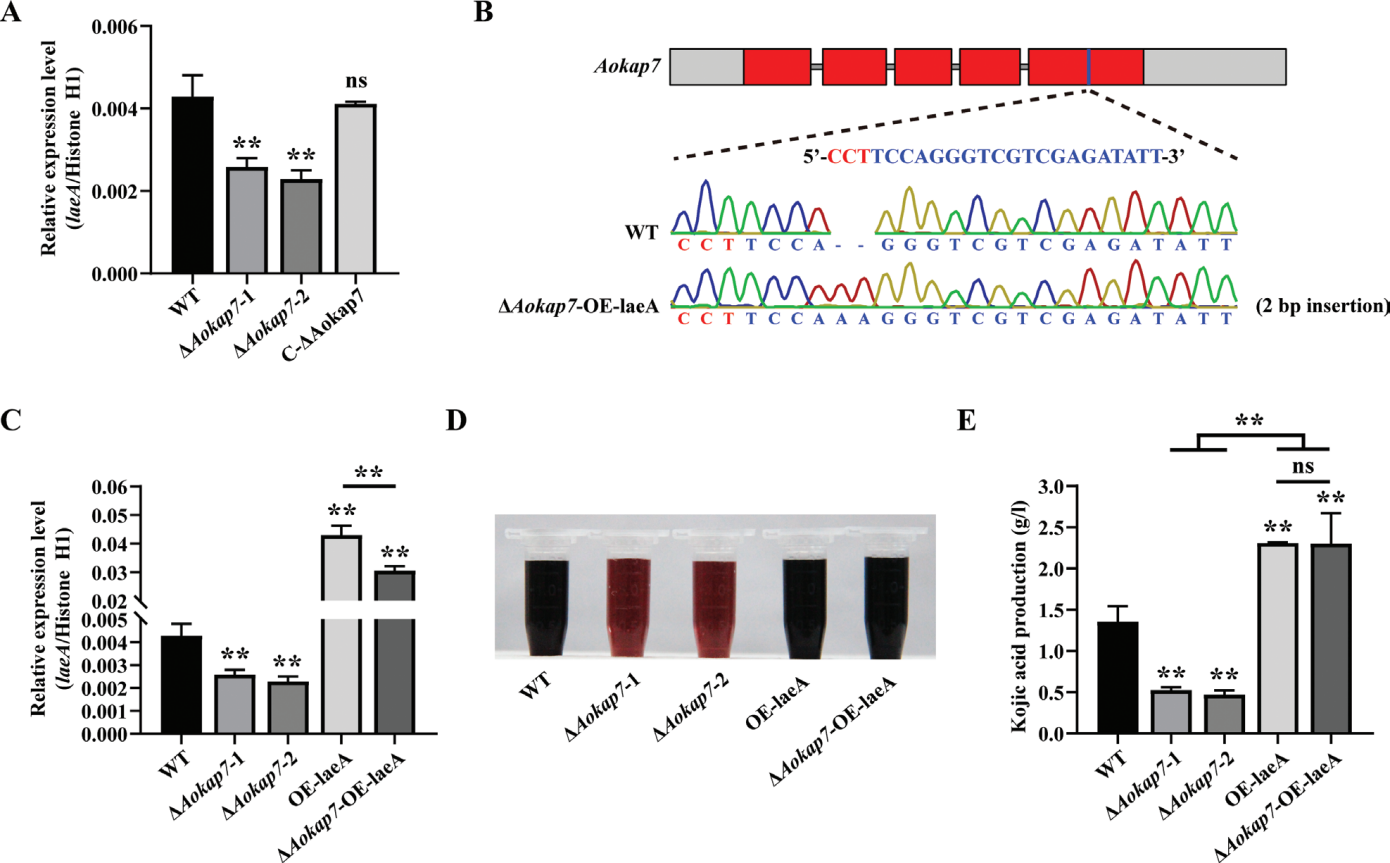
Influence of *laeA* overexpression in the Δ*Aokap7* strain background on kojic acid synthesis. **A** Expression proﬁle of *laeA* in the wild-type (WT), Δ*Aokap7*-1, Δ*Aokap7*-2 and C-Δ*Aokap7* after four days of fermentation; **B** Construction of the Δ*Aokap7*-OE-laeA strain with a 2-bp insertion in the *Aokap7* transcript. The target sequence for *Aokap7* is indicated in blue and its protospacer adjacent motif (PAM) is depicted in red; **C** The transcript level of *laeA* in the WT, *Aokap7* disruptants, *laeA* overexpression strain (OE-laeA) and Δ*Aokap7*-OE-laeA strain cultured in kojic acid liquid medium for four days; **D** Assessment of kojic acid production in the WT, Δ*Aokap7*-1, Δ*Aokap7*-2, OE-laeA and Δ*Aokap7*-OE-laeA strains cultivated in kojic acid liquid medium for seven days; **E** Quantitative analysis of kojic acid production in the WT, Δ*Aokap7*-1, Δ*Aokap7*-2, OE-laeA and Δ*Aokap7*-OE-laeA strains incubated in kojic acid liquid medium for seven days. Asterisks indicate statistically signiﬁcant differences compared to the WT strain based on Student’s t-tests (** *p* < 0.01; ns, no significant difference).

**Figure 8.**
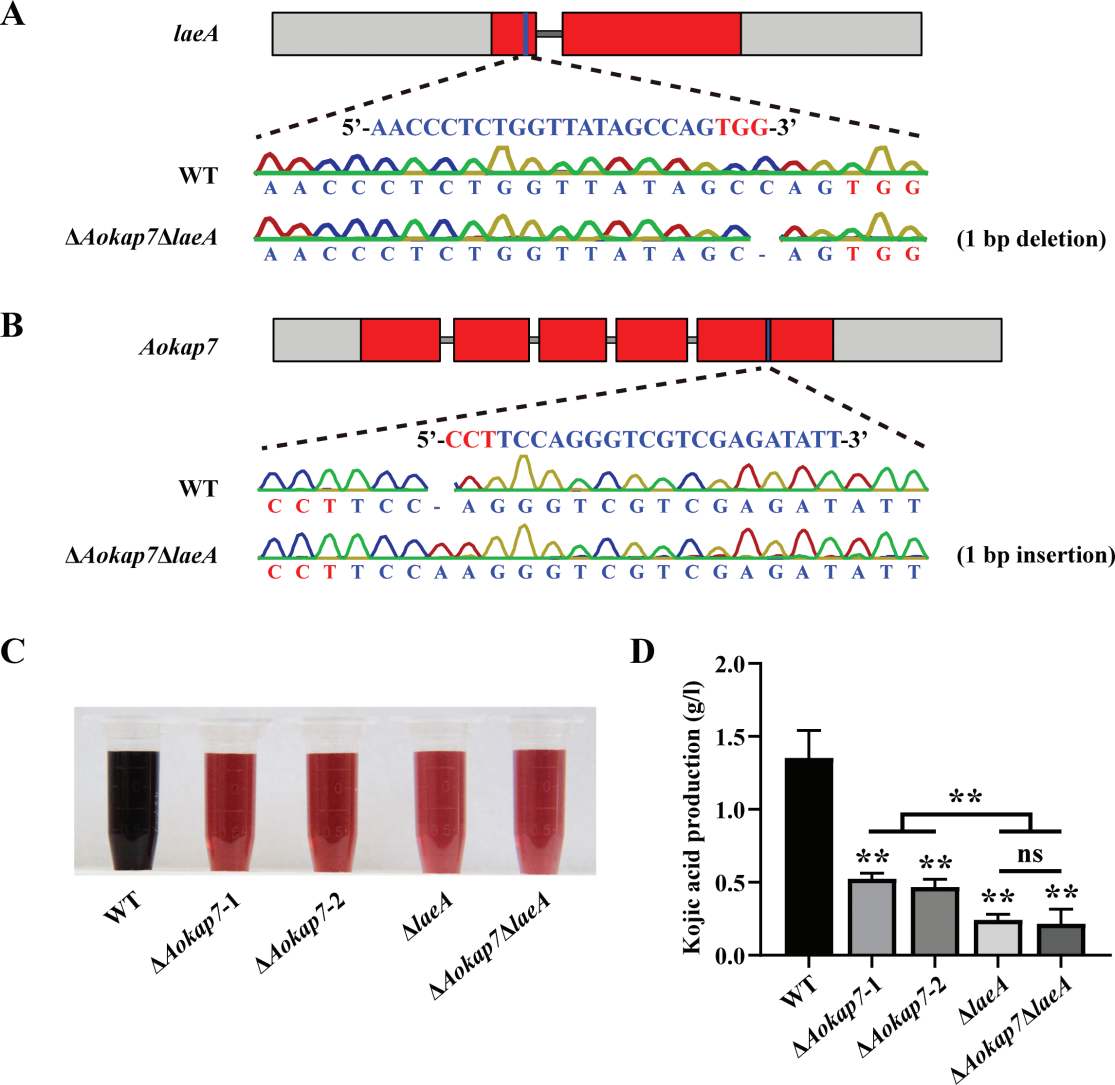
Effects of the loss of *laeA* in the Δ*Aokap7* strain on kojic acid production. **A, B** Generation of the double mutant of *laeA* and *Aokap7* (Δ*Aokap7*Δ*laeA*). Initially, the *laeA* gene was mutated by the CRISPR/Cas9 system to obtain the *laeA* disruptant; (A). Subsequently, the *Aokap7* gene in the *laeA* disruption strain was disrupted using the CRISPR/Cas9 technology; (B). The Δ*Aokap7*Δ*laeA* strain harboured a 1-bp insertion and a 1-bp deletion in the transcripts of *Aokap7* and *laeA*, respectively. The target sequence for *Aokap7* is indicated in blue and its protospacer adjacent motif (PAM) is indicated in red; **C** Visualisation of the colorimetric reaction of kojic acid produced by the wild-type (WT), Δ*Aokap7*-1, Δ*Aokap7*-2, Δ*laeA* and Δ*Aokap7*Δ*laeA* strains following fermentation for seven days in kojic acid liquid medium; **D** Quantification of kojic acid yield from the WT, Δ*Aokap7*-1, Δ*Aokap7*-2, Δ*laeA* and Δ*Aokap7*Δ*laeA* strains cultivated in kojic acid liquid medium for seven days. Asterisks indicate statistically signiﬁcant differences compared to the WT strain, based on Student’s t-tests (** *p* < 0.01; ns, no significant difference).

The aforementioned data indicate that *Aokap7* works upstream of *kojR*. Notably, KojR is under the control of the global regulator LaeA. Subsequently, an exploration into the interplay between *Aokap7* and *laeA* was conducted to elucidate how *Aokap7* affects kojic acid production. Initially, the expression of *laeA* in the *Aokap7* disruption strain was analysed by qPCR. The result revealed that the mRNA level of *laeA* was significantly down-regulated upon *Aokap7* disruption (Fig. 7A), suggesting a putative positive regulatory role of *Aokap7* on LaeA in the context of kojic acid biosynthesis. Subsequently, we postulated that the diminished kojic acid yield in the Δ*Aokap7* strain could be attenuated by overexpressing *laeA*. To address this hypothesis, we constructed the Δ*Aokap7*-OE-laeA strain, where *Aokap7* contained a 2-bp insertion and *laeA* was overexpressed using *A. oryzae amyB* promoter (Fig. 7B, C). Following a seven-day cultivation in kojic acid liquid medium, the quantification of kojic acid revealed that the Δ*Aokap7*-OE-laeA strain had comparable levels of kojic acid production to the *laeA* overexpression strain (Fig. 7D, E), suggesting that *Aokap7* plays a role upstream of *laeA*. To further confirm the genetic relationship between *Aokap7* and *laeA*, we constructed the Δ*Aokap7*Δ*laeA* double disruptant (Suppl. material 1: fig. S3), where *Aokap7* and *laeA* harboured a 1-bp insertion and a 1-bp deletion, respectively (Fig. 8A, B). The kojic acid production of the Δ*Aokap7*Δ*laeA* double mutant paralleled that of the *laeA*-disrupted mutant (Fig. 8C, D). These results indicated that *Aokap7* regulated kojic acid production through *laeA*.

### ﻿*Aokap7* genetically acts downstream of *AozfA* to regulate kojic acid production

**Figure 9.**
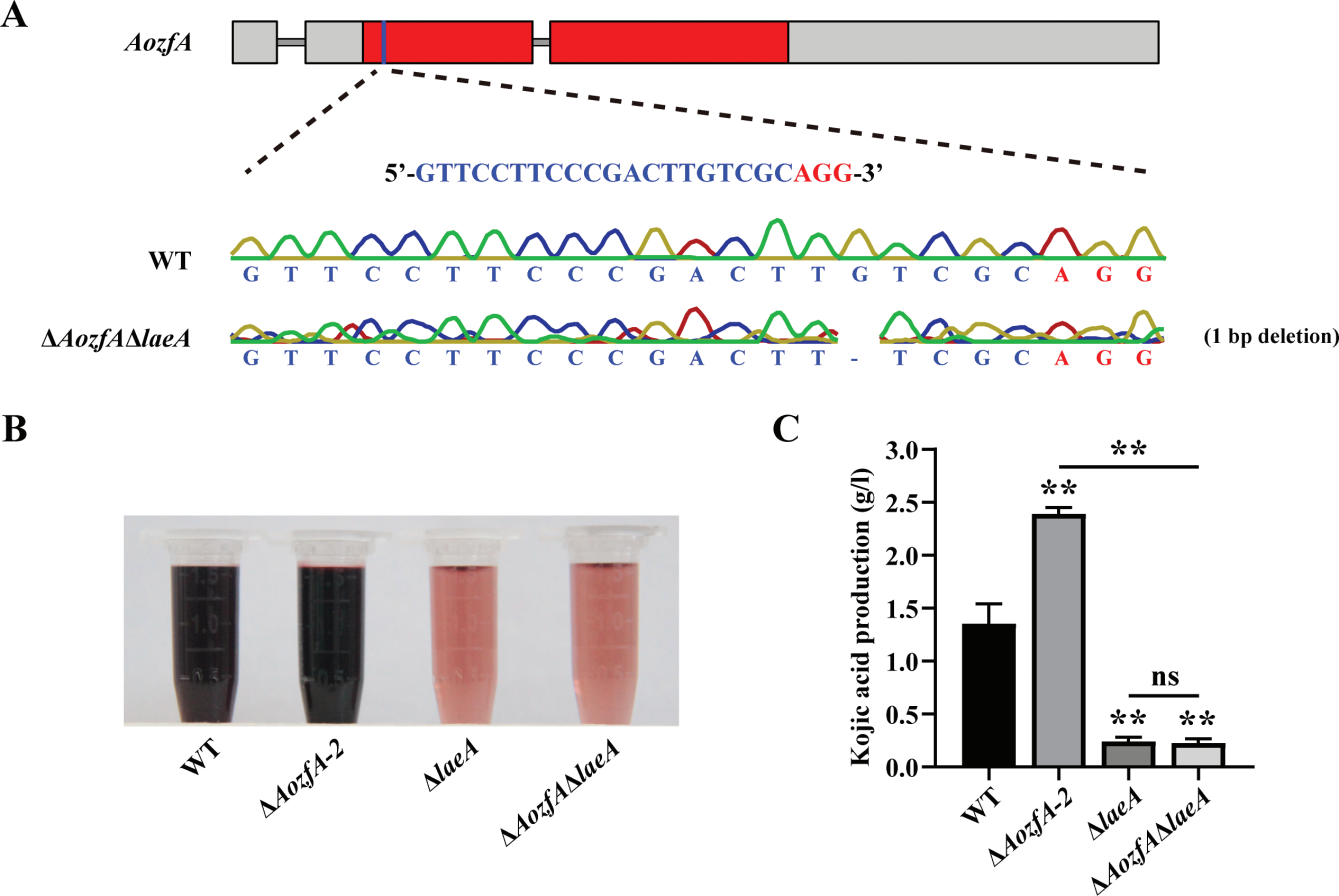
Disruption of *laeA* in *AozfA*-disrupted mutant inhibited kojic acid production. **A** The double mutant Δ*AozfA*Δ*laeA* was constructed using the CRISPR/Cas9 system, based on the *laeA*-disrupted mutant. The Cas9 targeted sequence for *AozfA* is shown in blue and the protospacer adjacent motif (PAM) sequence is highlighted in red; **B** Colorimetric assessment of kojic acid production by the wild-type (WT), *AozfA*-disrupted mutant (Δ*AozfA*-2), *laeA*-disrupted strain (Δ*laeA*) and double mutant of *laeA* and *AozfA* (Δ*AozfA*Δ*laeA*) after cultivation in kojic acid liquid medium for seven days at 200 rpm and 30 °C; **C** Quantification of kojic acid produced by the WT, ∆*AozfA*-2, ∆*laeA* and ∆*AozfA*∆*laeA* strains following a seven-day incubation in kojic acid liquid medium at 30 °C. ** *p* < 0.01 represents significant differences between the WT and mutant strains, while ‘ns’ indicates no significant difference.

**Figure 10.**
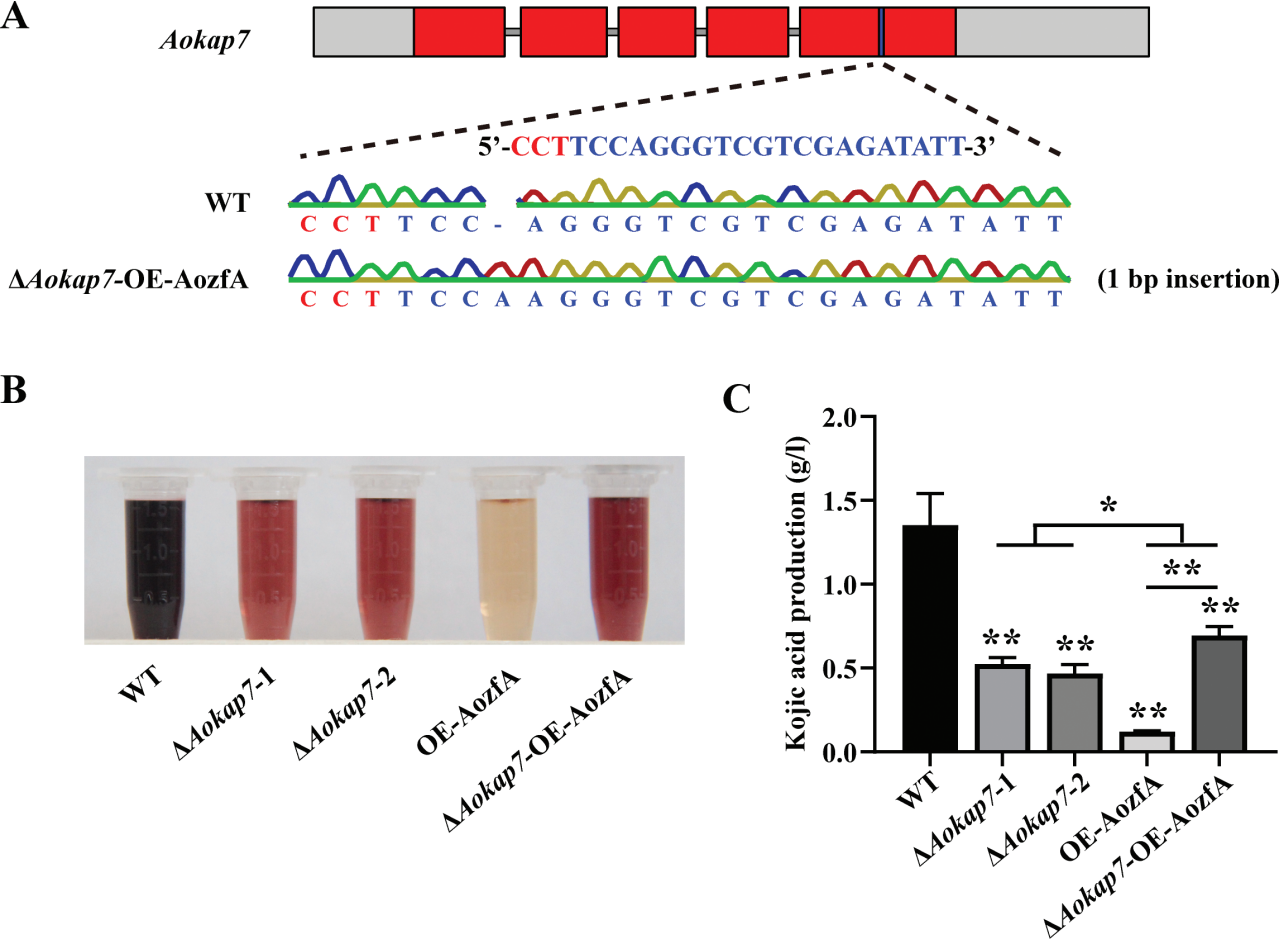
Disruption of *Aokap7* in the *AozfA* overexpression strain rescued the declined kojic acid production. **A** A targeted mutation of *Aokap7* in the *AozfA* overexpression strain was achieved using the CRISPR/Cas9 technology, resulting in the creation of the Δ*Aokap7*-OE-AozfA strain with a 1-bp insertion in the *Aokap7* transcript. The targeted sequence of *Aokap7* along with its protospacer adjacent motif (PAM) are highlighted in blue and red, respectively; **B** Visualisation of kojic acid through colour reactions with ferric ions was conducted for the wild-type (WT), Δ*Aokap7*-1, Δ*Aokap7*-2, OE-AozfA and Δ*Aokap7*-OE-AozfA strains cultivated in kojic acid liquid medium for seven days; **C** The yield of kojic acid from the WT, Δ*Aokap7*-1, Δ*Aokap7*-2, OE-AozfA and Δ*Aokap7*-OE-AozfA strains was quantified. Statistical significance denoted by * *p* < 0.05 and ** *p* < 0.01 represents significant differences between the WT and mutant strains.

**Figure 11.**
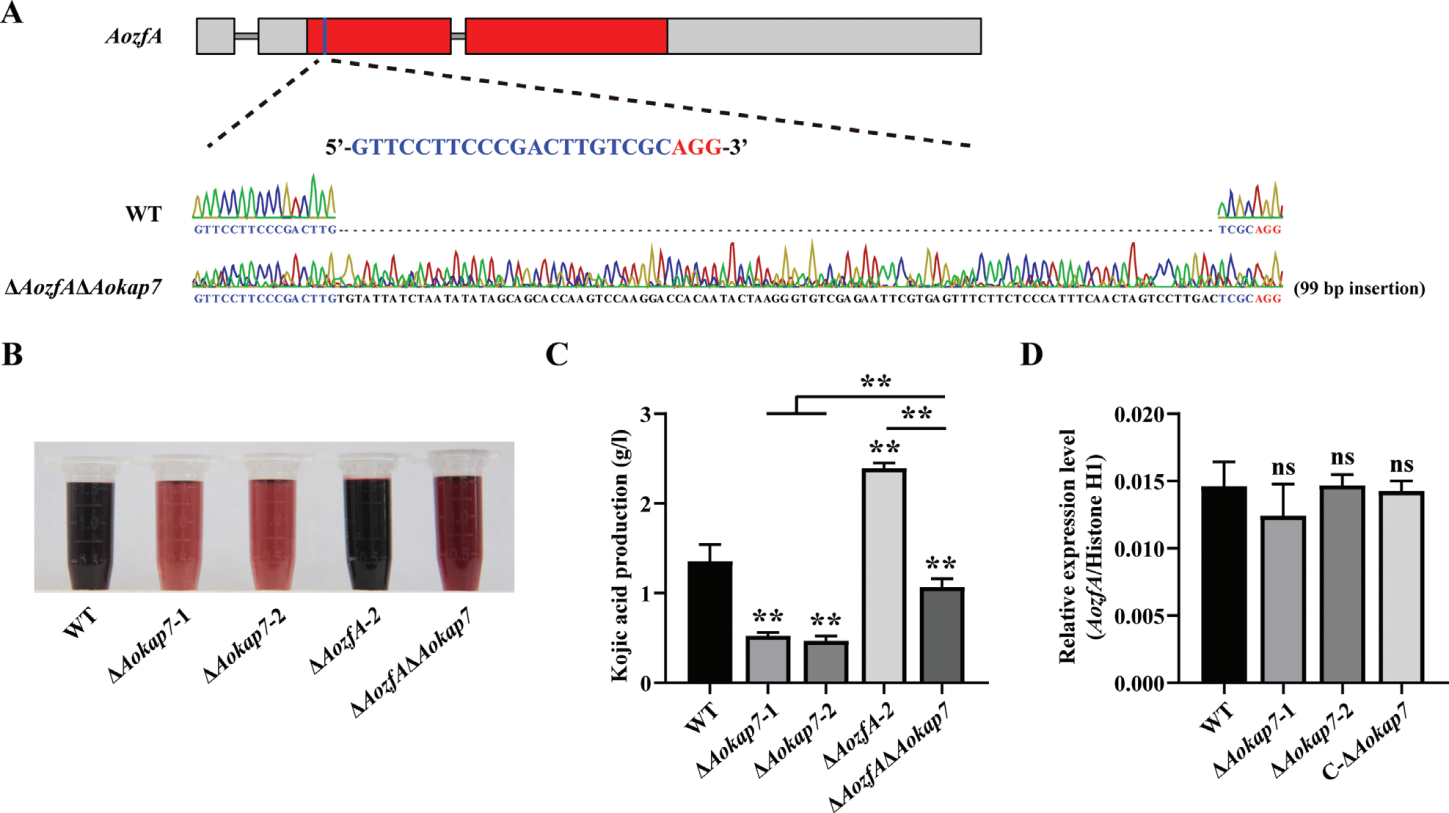
Effects of *Aokap7* disruption in the *AozfA*-disrupted strain on kojic acid production. **A** A double mutant, Δ*AozfA*Δ*Aokap7*, was constructed from the Δ*Aokap7*-1 mutant via the CRISPR/Cas9 system. The Δ*AozfA*Δ*Aokap7* strain harboured a 99-bp insertion in the *AozfA* transcript. The target sequence in *AozfA* is highlighted in blue and the PAM is shown in red; **B** Colorimetric reactions indicating kojic acid production were observed in the wild-type (WT), Δ*Aokap7*-1, Δ*Aokap7*-2, Δ*AozfA*-2 and Δ*AozfA*Δ*Aokap7* strains after seven days of cultivation in kojic acid liquid medium; **C** Quantification of kojic acid production in the WT, Δ*Aokap7*-1, Δ*Aokap7*-2, Δ*AozfA*-2 and Δ*AozfA*Δ*Aokap7* strains after seven days of cultivation in the kojic acid liquid medium was performed; **D** The expression levels of *AozfA* were assessed across in the WT, Δ*Aokap7*-1, Δ*Aokap7*-2 and the *Aokap7* complemented strain. Statistical significance denoted by ** *p* < 0.01 highlights noteworthy differences between the WT and the mutants, ‘ns’ indicates no significant different.

Previous research has established that *AozfA* negatively regulates kojic acid synthesis through *kojR*. Our current findings further elucidate that *Aokap7* functions upstream of *laeA* in the regulation of kojic acid production. To elucidate the relationship between *Aokap7* and *AozfA*, we firstly explored the genetic interactions between *AozfA* and *laeA* by targeted mutagenesis of *AozfA* in the *laeA* disruptant background using the CRISPR/Cas9 technology. The double mutant of *AozfA* and *laeA* (Δ*AozfA*Δ*laeA*) was successfully obtained for analysis of kojic acid production (Fig. 9A). We measured the yield of kojic acid in the WT, Δ*AozfA*-2, Δ*laeA*, and Δ*AozfA*Δ*laeA* strains, and found that the kojic acid production in the Δ*AozfA*Δ*laeA* double mutant was decreased compared with Δ*AozfA*-2, paralleling the levels observed in Δ*laeA* (Fig. 9B, C), indicating that *AozfA* functions upstream of *laeA* in the regulation of kojic acid synthesis. Subsequently, we created the Δ*AozfA*Δ*Aokap7* double mutant and Δ*Aokap7*-OE-AozfA that featured overexpression of *AozfA* and disruption of *Aokap7* (Figs 10A, 11A). After a seven-day fermentation in kojic acid liquid medium, the Δ*Aokap7*-OE-AozfA strain demonstrated an increased yield of kojic acid relative to the *AozfA* overexpression strain, and notably higher levels than in the *Aokap7* disruptants (Fig. 10B, C). Conversely, the Δ*AozfA*Δ*Aokap7* strain displayed reduced kojic acid production compared with the Δ*AozfA*-2 strain, but greater production compared to the *Aokap7* disrupted strains (Fig. 11B, C). Furthermore, we evaluated *AozfA* transcript levels in the WT, *Aokap7* disruptants and C-Δ*Aokap7* strain. RT-qPCR analysis revealed no significant changes in *AozfA* expression across these strains (Fig. 11D). Collectively, our findings support the positioning of *Aokap7* downstream of *AozfA* in the regulatory framework governing kojic acid biosynthesis.

### ﻿*Aokap7* modulates oxidative stress response by regulating ROS scavenging genes

**Figure 12.**
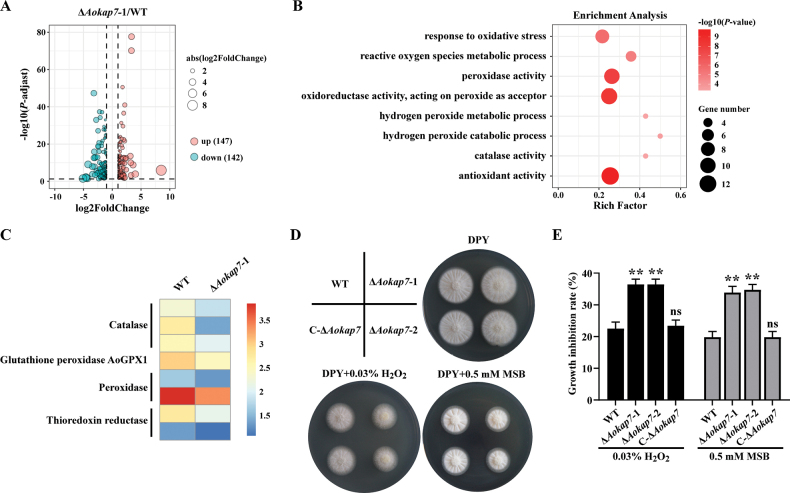
Differentially expressed genes between the wild-type (WT) and *Aokap7*-disrupted strains by transcriptomic analysis. **A** Volcano plots showing differentially expressed genes in the WT and *Aokap7* disruption strains. The green and red represent down-regulated genes and up-regulated genes, respectively; **B** Gene Ontology (GO) analysis of 289 differentially expressed genes in the WT and *Aokap7* disruptant; **C** Heatmap of differentially expressed genes involved in scavenging reactive oxygen species (ROS) in GO analysis; **D** Colony morphology of the WT, *Aokap7* disruptants cultured on DPY agar plates supplemented with 0.03% H_2_O_2_ or 0.5 mM MSB for two days; **E** The growth inhibition rate of the WT, *Aokap7* disruptants and the complemented strain grown on DPY plates with H_2_O_2_ or MSB. Statistical significance denoted by **p < 0.01 when compared to the WT strain; “ns” indicates no significant difference.

To obtain a general overview of the function of *Aokap7*, we conducted transcriptomic analysis of the WT and Δ*Aokap7*-1 strains cultured in kojic acid liquid medium for three days. Differential expression analysis revealed 147 up-regulated and 142 down-regulated genes in the Δ*Aokap7*-1 mutant (Fig. 12A). Gene Ontology (GO) enrichment indicated significant associations with oxidative stress response (GO:0006979), reactive oxygen species (ROS) metabolism (GO:0072593), and hydrogen peroxide catabolism (GO:0042744) (Fig. 12B). Notably, eight ROS scavenging-related genes encoding catalase (CAT), glutathione peroxidase (GPX), peroxidase (POD) and thioredoxin reductase (TRXR), were down-regulated significantly (Fig. 12C). To assess oxidative stress tolerance, the WT, *Aokap7* disruptants and complemented strain were exposed to 0.03% H_2_O_2_ or 0.5 mM MSB. After cultivation for two days, the *Aokap7* disruption strains exhibited significantly higher growth inhibition compared to the WT and complemented strains (Fig. 12D, E). These findings demonstrate that *Aokap7* disruption impairs ROS detoxification by suppressing scavenging gene expression, thereby compromising antioxidant capacity in *A. oryzae*.

### ﻿Disruption of the *Aokap7* gene increases the sensitivity of *A. oryzae* to heat, cell-wall, and NaCl-mediated osmotic stresses

**Figure 13.**
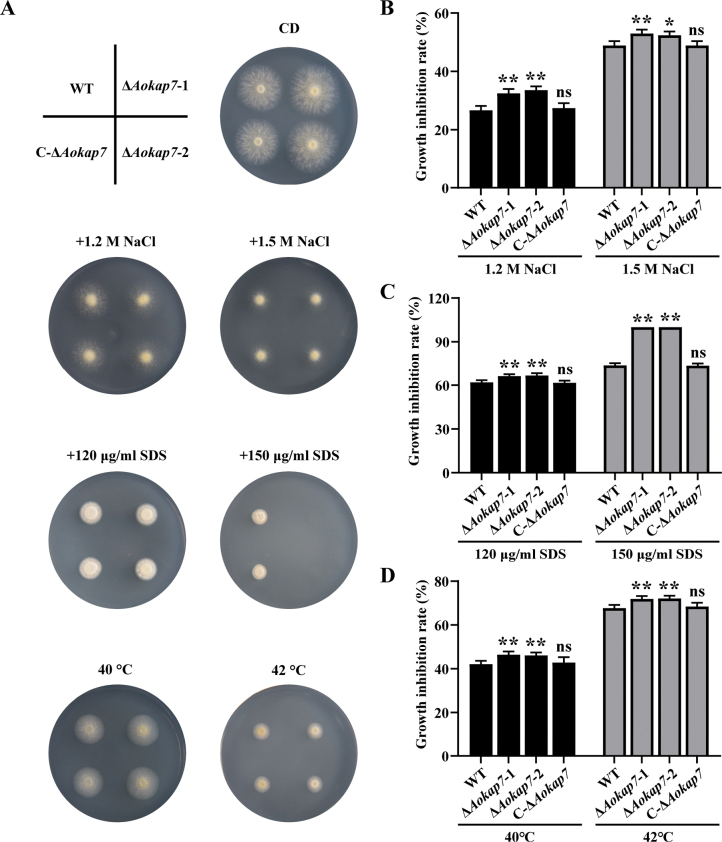
Involvement of *AoKap7* in the response to environmental stresses. **A** Colony morphology of the wild-type (WT), Δ*Aokap7*-1, Δ*Aokap7*-2 and C-Δ*Aokap7* strains cultured on CD agar plates supplemented with 1.2/1.5 M NaCl, 120/150 µg/ml SDS or incubated at 40/42 °C for three days; **B–D** Growth inhibition rates of the WT, Δ*Aokap7*-1, Δ*Aokap7*-2 and C-Δ*Aokap7* strains on CD plates with NaCl (B), SDS (C) or incubated at 40/42 °C (D). Statistical significance is indicated by * *p* < 0.05 and **p < 0.01 when compared to the WT strain; “ns” indicates no significant difference.

To investigate the role of *Aokap7* in the response of *A. oryzae* to other stresses, we subjected the WT, *Aokap7* disruptants and *Aokap7* complemented strain to heat, cell-wall (SDS) and osmotic (NaCl) treatments. The strains were inoculated on to CD medium supplemented with 1.2/1.5 M NaCl or 120/150 µg/ml SDS, or incubated at 40/42 °C to assess the effects of *Aokap7* disruption on the environmental response (Fig. 13A). After incubation for three days, the growth inhibition rates of fungal colonies were analysed (Fig. 13B–D). The results showed that, under heat, cell-wall integrity, and osmotic stresses, the *Aokap7* disruption strains exhibited a significantly higher growth inhibition rate compared to the WT strain (Fig. 13B–D). These results suggest that the absence of *Aokap7* enhances the sensitivity of *A. oryzae* to heat, cell-wall, and NaCl-mediated osmotic stresses.

### ﻿Identification of *Aokap7*-binding motifs

**Figure 14.**
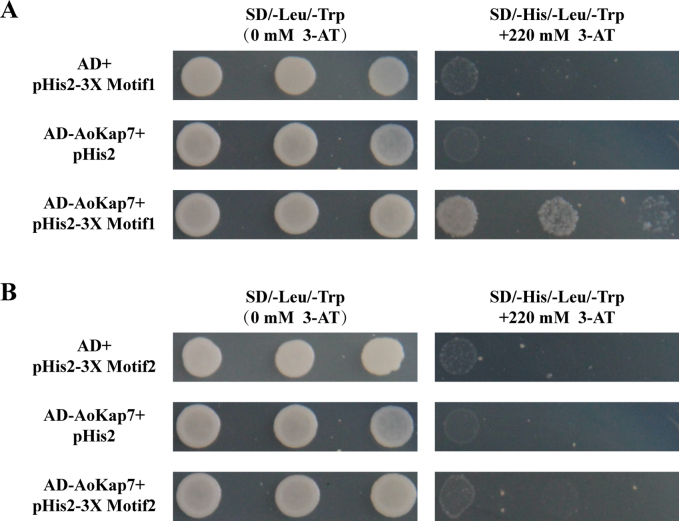
Yeast one-hybrid analysis of the motifs recognized by *AoKap7*. Three tandem copies of the Motif1 (A) or Motif2 (B) were cloned into pHIS2, forming pHIS2-3X Motif1 and pHIS2-3X Motif2 vectors, respectively. The coding sequence of *Aokap7* was inserted into pGADT7, generating AD-*Aokap7* plasmid. After co-transformation, transformed yeast colonies were spotted on SD/-Leu/–Trp and SD/-His/-Leu/–Trp/3-AT plates.

To determine the DNA sequences recognised by *Aokap7*, we generated a random 7-nucleotide insertion library in the Y187 yeast strain using the pHIS2 vector. This library was screened with the bait plasmid AD-*Aokap7* under stringent selection (SD/–His/–Leu/–Trp + 220 mM 3-AT). Sequencing of the pHIS2 plasmids revealed two candidate motifs: CGGCTCGG (Motif1) and CCCTCAC (Motif2). To validate specificity, we cloned three tandem repeats of each motif into pHIS2 and performed yeast one-hybrid (Y1H) assays. Only yeast strains carrying *Aokap7* with Motif1 exhibited robust growth (Fig. 14A, B), confirming that *Aokap7* directly binds to Motif1, but not Motif2.

### ﻿*Aokap7* regulates oxidative tolerance by directly binding to the *AoGPX1* promoter

**Figure 15.**
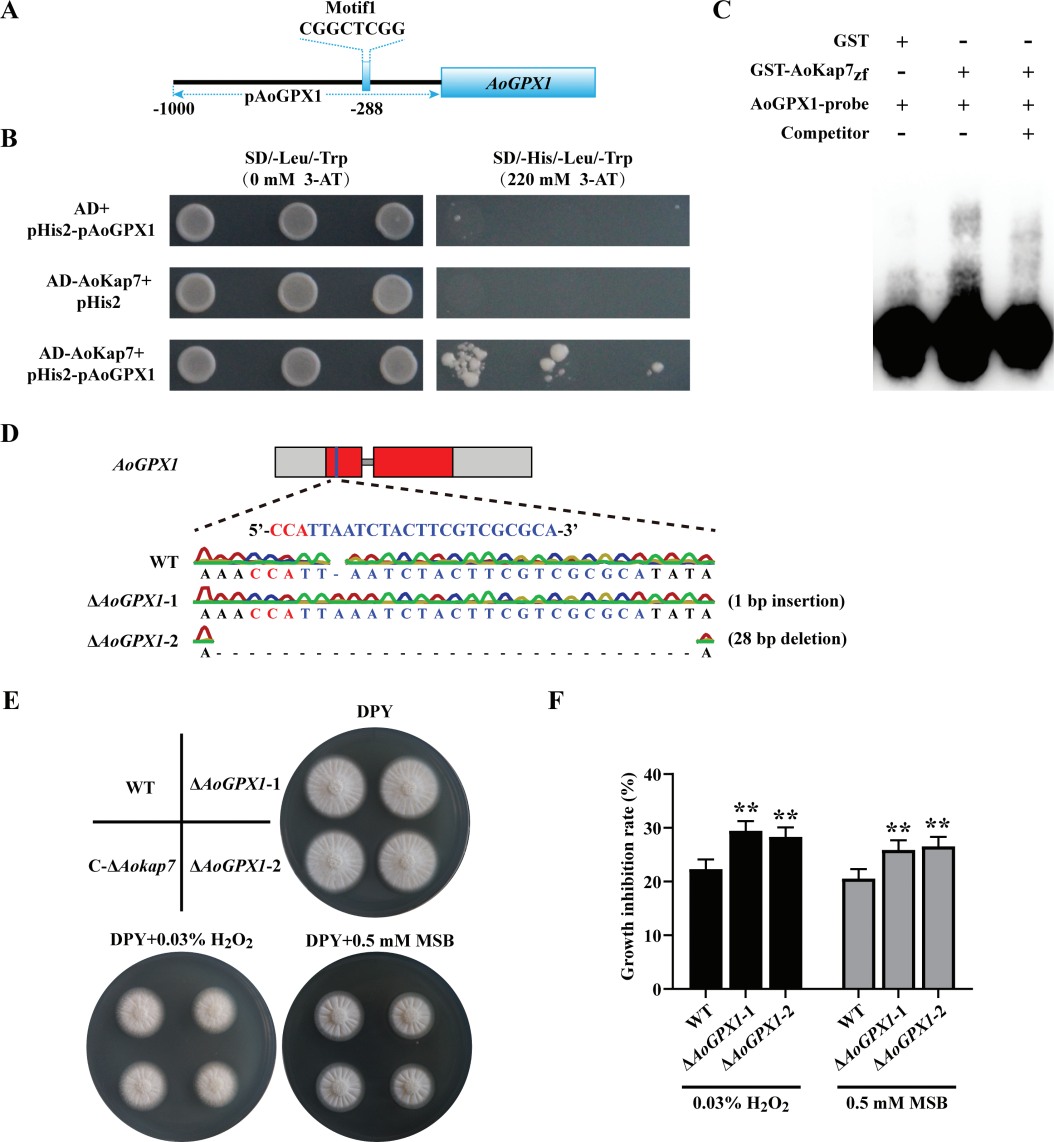
*AoKap7* directly bind to the *AoGPX1* promoter. **A** Schematic showing the promoter of *AoGPX1*. The small rectangular block indicates the Motif1 (CGGCTCGG) cis-elements. The 1,000-bp upstream sequence of the start codon of *AoGPX1* was designed as the *AoGPX1* promoter (pAoGPX1); **B** Yeast one-hybrid analysis of the binding of the *AoGPX1* promoter to *AoKap7*; **C**EMSA of *AoKap7* binding to the Motif1 element in the *AoGPX1* promoter in vitro. The zinc finger domain of *AoKap7* was fused with GST to generate GST-*Aokap7*_zf_. Biotin-labelled *AoGPX1* probes containing the Motif1 CGGCTCGG were incubated with puriﬁed GST or GST-*Aokap7*_zf_. Non-labelled probes at 200-fold higher concentrations were used for the competition assay; **D** Creation of the *AoGPX1* disruption mutants using the CRISPR/Cas9 system. The two *AoGPX1* disrupted mutants Δ*AoGPX1*-1 and Δ*AoGPX1*-2 were obtained, which harboured a 1-bp insertion and a 28-bp deletion in the *AoGPX1* gene, respectively. The sgRNA targeting *AoGPX1* is indicated in blue and the PAM is highlighted in red; **E** The phenotypes of the wild-type (WT), Δ*AoGPX1*-1, Δ*AoGPX1*-2 and C-Δ*Aokap7* strains grown on DPY agar medium with and without 0.03% H_2_O_2_ or 0.5 mM MSB for two days; **F** Growth inhibition rates of the WT, Δ*AoGPX1*-1, Δ*AoGPX1*-2 and C-Δ*Aokap7* strains following treatment with H_2_O_2_ or MSB. Statistical significance denoted by **p < 0.01 when compared to the WT strain.

Our findings demonstrate that *Aokap7* regulates target genes through recognition of Motif1 (CGGCTCGG) and modulates ROS scavenging-related gene expression. Thus, we searched for the Motif1 in the promoters of the ROS scavenging-related genes. Interestingly, one Motif1 was found in the promoter of *AoGPX1* (Fig. 15A). The promoter of *AoGPX1* containing the Motif1 was inserted into pHIS2 vector and used for Y1H analysis. Y1H assays confirmed *Aokap7* binding to the *AoGPX1* promoter (Fig. 15B). To further validate this interaction, we performed electrophoretic mobility shift assay (EMSA) using a GST-tagged zinc finger domain of *Aokap7* (residues 49–91, GST-*Aokap7*_zf_). Incubation of GST-*Aokap7*_zf_ with a labelled *AoGPX1* probe containing Motif1 resulted in a mobility shift, which was competitively inhibited by the unlabelled probe (Fig. 15C). The ability of *Aokap7* to bind the *AoGPX1* promoter was inhibited by the addition of the unlabelled probe (Fig. 15C). These results establish that *Aokap7* directly interacts with the *AoGPX1* promoter. To confirm that *AoGPX1* is indeed a direct target of *Aokap7*, we used the CRISPR/Cas9 technology to introduce mutations in the *AoGPX1* gene. Sequence analysis detected a 1-bp insertion and a 28-bp deletion in the transcripts of *AoGPX1*-disrupted mutants, Δ*AoGPX1*-1 and Δ*AoGPX1*-2, respectively (Fig. 15D). We subsequently assessed the sensitivity of these disruptants to oxidative stress, finding that the *AoGPX1* disruption strains exhibited heightened sensitivity to H_2_O_2_ and MSB compared to the WT strain (Fig. 15E, F).

## ﻿Discussion

*A. oryzae* harbours the predicted kojic acid gene cluster containing 13 genes according to comparative genomics of *A. nidulans*, *A. fumigatus* and *A. oryzae* (Terabayashi et al. 2010). Noteworthy, research has confirmed the pivotal function of the kojic acid gene cluster in the kojic acid synthesis of *A. oryzae* (Oda et al. 2011; Arakawa et al. 2019; Kudo et al. 2021; Chen et al. 2022b; Li et al. 2023; Chen et al. 2023). However, the specific roles of genes encompassed by the putative gene cluster remain ambiguous, with the exception of *kojA*, *kojR*, *kojT*, *Aokap4* and *Aokap6* (Terabayashi et al. 2010; Chen et al. 2022a; Chen et al. 2022b). In this current study, we identified and characterised the *Aokap7* gene near the kojic acid gene cluster and revealed that it encodes a novel zinc finger regulator involved in growth and kojic acid synthesis in *A. oryzae*. The production of secondary metabolism is closely associated with fungal development, often linked to mycelium growth and conidiation (Calvo and Cary 2015). Herein, loss of function in *Aokap7* led to the increase in spore germination, mycelium growth and conidia formation, but the reduction in kojic acid production (Figs 3, 4), indicating that *Aokap7* acts as an indispensable regulator of both growth and kojic acid synthesis in *A. oryzae*, which is supported by the observation that overexpression of *Aokap7* inhibited conidia formation and kojic acid synthesis although disruption and overexpression of *Aokap7* did not show opposite phenotypes, except for spore numbers (Figs 3, 4, Suppl. material 1: figs S5, S6). Furthermore, loss of function in the *Aokap7* gene accelerated spore germination, correlating with increased mRNA levels of *pkaB*, *rasA*, *mpkA* and *treB* (Fig. 3G–L). This suggests that *Aokap7* is required for conidial germination, potentially linked to cAMP and RasA signalling pathways, as well as the rapid degradation of intracellular trehalose mediated by the neutral trehalase, TreB. Notably, disruption of *Aokap4* or *Aokap6* near the kojic acid gene cluster also contributes to mycelium growth and conidia production, whereas their malfunction led to diminished kojic acid output (Chen et al. 2022a; Chen et al. 2022b). Given *Aokap4*, *Aokap6* and *Aokap7* are located near the kojic acid gene cluster, the resemblances in their impacts on growth and kojic acid biosynthesis suggest a non-random phenomenon. These findings bolster the hypothesis that genes neighbouring the kojic acid gene cluster undergo selective pressures, aiding *A. oryzae* in kojic acid production while inhibiting growth. This phenomenon is partly elucidated by the energy-intensive nature of kojic acid biosynthesis as a secondary metabolic process, initiated solely upon necessity (Barrios-González 2018).

**Figure 16.**
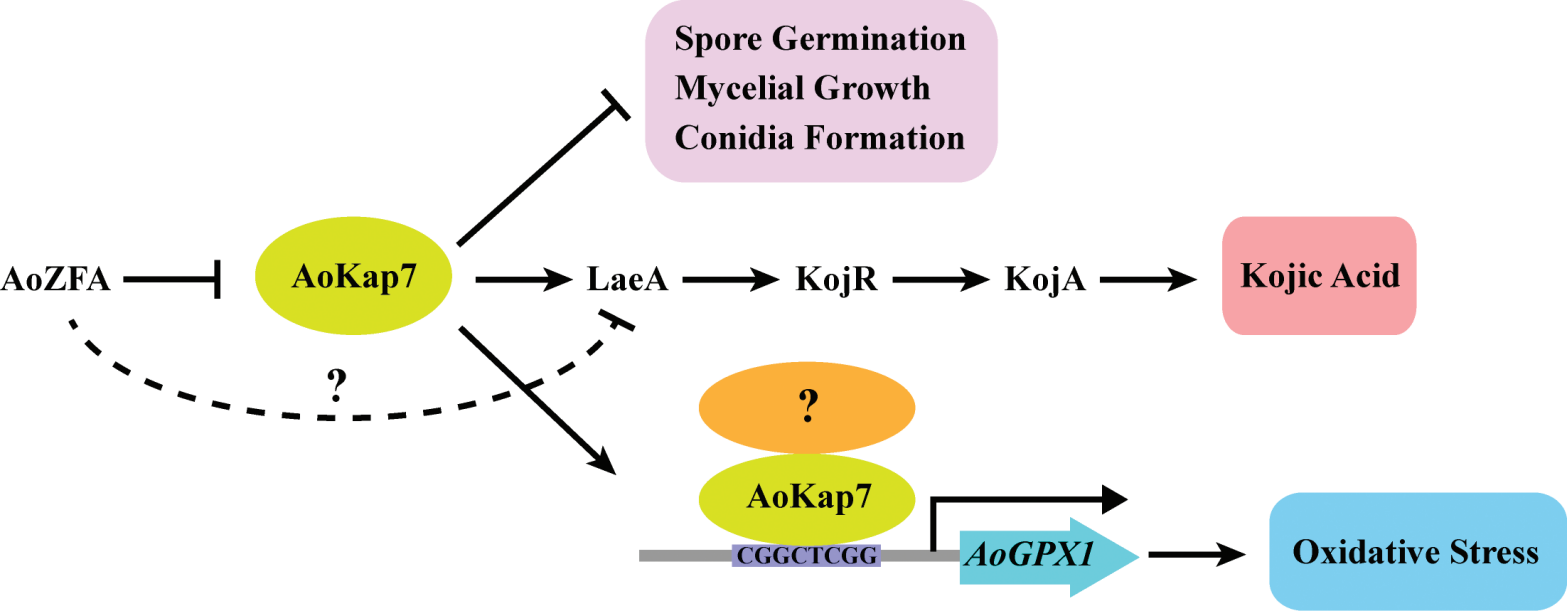
Schematic representation illustrating the functional role of *AoKap7* in the growth, oxidative stress and kojic acid production in *A. oryzae*. *AoKap7* negatively regulates the growth of *A. oryzae*, including spore germination, mycelium growth and conidial formation. A critical regulatory pathway governing kojic acid synthesis is identified, proceeding from AoZFA to LaeA to KojR. *AoKap7* mediates kojic acid production through the regulatory framework of LaeA and KojR. Notably, AoZFA acts upstream of *AoKap7*, also negatively regulating *laeA* through yet unidentified pathways, thereby influencing kojic acid production. Furthermore, *AoKap7* regulates oxidative tolerance by directly binding to the *AoGPX1* promoter.

Within the kojic acid gene cluster, KojR, a pivotal regulator, positively mediates kojic acid production through modulating *kojA* and *kojT* (Marui et al. 2011). In the present study, loss of *Aokap7* led to a decrease in kojic acid production, which is accompanied by declined expression levels of *kojR* and *kojA* (Fig. 4). Furthermore, overexpression of *kojR* in the *Aokap7*-disrupted mutant reversed the reduction in kojic acid production, whereas disruption of *kojR* in the *Aokap7* disruptant background led to the Δ*kojR*-like phenotype in kojic acid production (Figs 5, 6), suggesting that *Aokap7* regulates kojic acid production through KojR. Furthermore, the global regulator LaeA regulates kojic acid synthesis through influencing the expression of *kojR*, *kojA* and *kojT* (Oda et al. 2011). Here, we observed that either overexpression or disruption of *laeA* in the *Aokap7* disruption strain caused a comparable impact on kojic acid synthesis as manipulating *kojR* in the *Aokap7* disruption strain background (Figs 5–8). These findings suggest that *Aokap7* mediates kojic acid synthesis through the LaeA-KojR regulatory pathway (Fig. 16). Additionally, we demonstrated that the zinc finger regulator AoZFA negatively regulates kojic acid production through LaeA (Fig. 9). Interestingly, either mutation in *Aokap7* in the *AozfA* overexpression strain background or disruption of *Aokap7* in the *AozfA* disruption strain could reverse their kojic acid production (Figs 10, 11), indicating that *Aokap7* functions downstream of *AozfA*, which is further supported by the lack of changes in the *AozfA* expression in the WT, *Aokap7* mutants, and its complemented strains, as well as the growth similarities observed between the double mutant Δ*AozfA*Δ*Aokap7* and *Aokap7* disruptants, in contrast to Δ*AozfA*-2 (Fig. 11D and Suppl. material 1: fig. S4). However, disruption of *Aokap7* in the *AozfA* overexpression or disruption strains resulted in a higher yield of kojic acid relative to *Aokap7* disruptants (Figs 10, 11), implying that there is another pathway regulated by AoZFA via controlling LaeA (Fig. 16). Moreover, these findings suggest the existence of a pathway from AoZFA to LaeA to KojR involved in the regulatory mechanisms governing kojic acid production and *Aokap7* is under the control of AoZFA and regulated kojic acid synthesis through the LaeA-KojR module (Fig. 16). In *A. niger*, *nrkB* has been identified as the homologue of *Aokap7*, and disruption of *nrkB* increases kojic acid production (Wu et al. 2023), in sharp contrast to the effect observed with *Aokap7*. However, loss of the homologue of *AoKap4* or *Aokap6* in *A. niger* had no effect on kojic acid production (Wu et al. 2023), whereas disruption of *AoKap4* or *Aokap6* in *A. oryzae* decreased kojic acid production (Chen et al. 2022a; Chen et al. 2022b). Furthermore, both overexpression and disruption of *Aokap7* led to reduced kojic acid production, accompanied by decreased expression levels of *kojA* and *kojR* (Fig. 4 and Suppl. material 1: fig. S6). These findings indicate that the regulation mechanism of genes near the kojic acid gene cluster involved in kojic acid synthesis is complicated and the underlying regulatory mechanism of kojic acid synthesis varies between *A. oryzae* and *A. niger*.

A previous study has revealed that the histone deacetylase AoHst4 exerts a negative regulatory effect on kojic acid production by suppressing LaeA (Kawauchi et al. 2013). In our experiments, disruption of *Aokap7* led to reduced kojic acid production coupled with the declined expression of *laeA* (Fig. 7A). Intriguingly, overexpression of *laeA* could reverse the decreased yield of kojic acid (Fig. 7), underscoring the role of *Aokap7* in mediating kojic acid synthesis by activating LaeA. This interpretation is corroborated by the significant difference in *laeA* expression levels between the OE-laeA strain and the Δ*Aokap7*-OE-laeA strain (Fig. 7C), potentially attributed to the absence of background expression induced by *Aokap7* in the Δ*Aokap7*-OE-laeA strain. Given the involvement of AoHst4 in deactivating LaeA, there lies a possibility of *Aokap7* serving as a competitive mechanism to regulate LaeA against AoHst4. Further investigations are needed to estimate the relationship between *Aokap7* and AoHst4. Additionally, transcriptional activation assays indicated that the *Aokap7* protein lacks an activation domain (Fig. 2D), suggesting that the activation of downstream genes by *Aokap7* necessitates the involvement of an additional transcriptional activator (Fig. 16). Consequently, identifying proteins that interact with *Aokap7* will be essential for elucidating the mechanisms by which *Aokap7* is involved in growth, stress and kojic acid synthesis.

Previous studies on *A. flavus* and *A. parasiticus* have underscored the significant impact of oxidative stresses on kojic acid production (Chang et al. 2011; Tian et al. 2021). When these fungi were under oxidative stress, they produce more kojic acid (Chang et al. 2011; Zhang et al. 2014; Tian et al. 2021), indicating a positive correlation between oxidative stress and kojic acid. This is explained by the fact that kojic acid acts as an antioxidant to scavenge reactive oxygen species (Zhang et al. 2014; Tian et al. 2021). While the C_2_H_2_-type zinc-finger regulator Msn2 has been implicated in this process (Chang et al. 2011), its regulatory mechanism remains undefined. Our study reveals that both disruption and overexpression of *Aokap7* increases the sensitivity of *A. oryzae* to oxidative stress (Fig. 12 and Suppl. material 1: fig. S7). Notably, *Aokap7* disruption down-regulates key ROS detoxification genes, including *GPX*, *CAT* and *POD* (Fig. 12C) and demonstrates direct binding of *Aokap7* to the *AoGPX1* promoter. Furthermore, disruption of *AoGPX1* resulted in an increased sensitivity of *A. oryzae* to oxidative stress (Fig. 15E, F), indicating that *Aokap7* modulates oxidative tolerance by its interaction with the *AoGPX1* promoter. Additionally, *Aokap7* regulates kojic acid production by the LaeA-KojR regulatory module. These findings suggest that position *Aokap7* as a central regulator coordinating oxidative stress adaptation with kojic acid biosynthesis in *A. oryzae* (Fig. 16). While the *Aokap7*-binding motif has been identified, the specific target genes involved in kojic acid synthesis remain to be elucidated. Employing a combination of RNA sequencing and chromatin immunoprecipitation sequencing (ChIP-seq) will provide a comprehensive understanding of the functional roles of *Aokap7*.

In conclusion, our study identifies *Aokap7* as a novel zinc finger regulator that lacks transcriptional activation activity, and regulates fungal development (spore germination, hyphal growth, and conidiation) and oxidative stress response through direct binding to the *AoGPX1* promoter. Functioning downstream of AoZFA, *Aokap7* modulates kojic acid biosynthesis via the LaeA-KojR regulatory module. These findings not only bolster our comprehension of the kojic acid biosynthetic pathway, but also shed light on the coordinated roles of genes near the kojic acid gene cluster in the crosstalk between kojic acid production and oxidative stress adaptation, potentially paving the way for the discovery of regulatory network of kojic acid synthesis.

## ﻿Additional information

### Use of AI

No use of AI was reported.

### Adherence to national and international regulations

All the fungal strains used in this study have been legally obtained, respecting the Convention on Biological Diversity (Rio Convention).

### Author ORCIDs

Ting Qiu https://orcid.org/0009-0004-2561-0961

Ziming Chen https://orcid.org/0009-0005-2398-8466

Huanxin Zhang https://orcid.org/0000-0002-4048-4543

Lihua Yao https://orcid.org/0000-0002-4112-1920

Zhe Zhang https://orcid.org/0000-0003-0632-7672

### Data availability

All of the data that support the findings of this study are available in the main text or Supplementary Information.

## Supplementary materials

Supplementary material 1 Construction of *Aokap7* complemented strain and primers used in this study Data type docx Explanation note **fig. S1.** Construction of *AoКap7* complemented strain. **fig. S2.** Effects of disrupting *kojR* in *AoКap7* disrupted strain on growth. **fig. S3.** Effects of *laeA* disruption in *AoКap7* disrupted strain background on growth. **fig. S4.** Effects of *AozfA* disruption in *AoКap7*-disrupted strain background on growth. **fig. S5.** Effluences of *AoКap7* overexpression on growth. **fig. S6.** Effects of *AoКap7* overexpression on kojic acid production. **fig. S7.** Overexpressing *AoКap7* increases sensitivity of *A. oryzae* to oxidative stress. **table S1.** Primers used in this study. https://binary.pensoft.net/file/1424043This dataset is made available under the Open Database License (http://opendatacommons.org/licenses/odbl/1.0/). The Open Database License (ODbL) is a license agreement intended to allow users to freely share, modify, and use this Dataset while maintaining this same freedom for others, provided that the original source and author(s) are credited.Ting Qiu, Ziming Chen, Ziming Chen, Bangfu Deng, Lihua Yao, Zhe Zhang
