# Supplementary material for: ﻿The Zn(II)2-Cys6-type zinc finger protein AoKap7 is involved in the growth, oxidative stress and kojic acid synthesis in Aspergillus oryzae
Source: IMA Fungus. 2025 Sep 25;16:e153994. doi: 10.3897/imafungus.16.153994 (PMC12491976; doi:10.3897/imafungus.16.153994)
Supplement: Supplementary material 1 — Construction of Aokap7 complemented strain and primers used in this study [file imafungus-16-e153994-s001.docx]

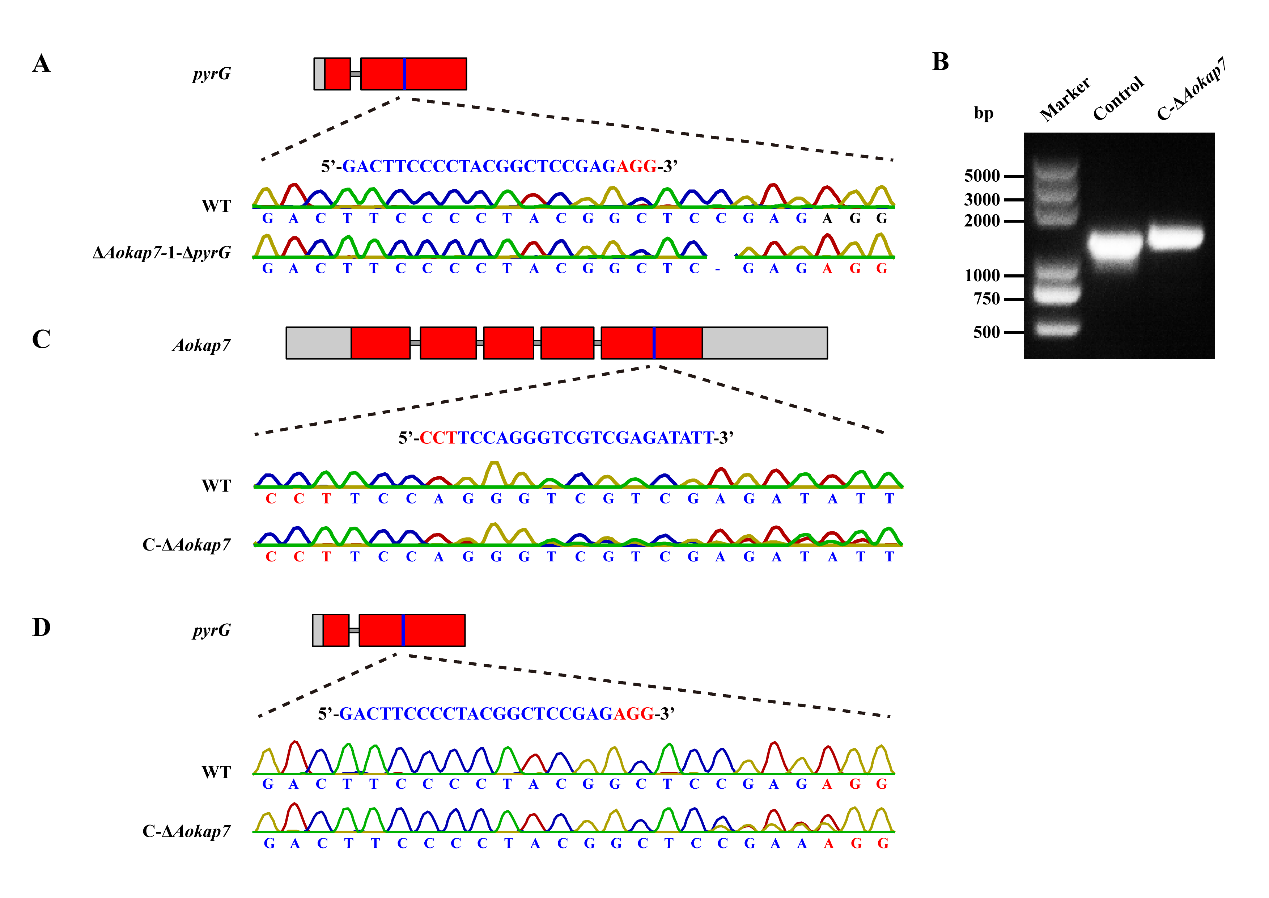


**Figure S1.** Construction of *Aokap7* complemented strain. (A) The *pyrG* gene in the Δ*Aokap7*-1 was disrupted by the CRISPR/Cas9 system, resulting in the creation of the Δ*Aokap7*-1-Δ*pyrG* strain with a knockout of the *pyrG* gene. The Δ*Aokap7*-1-Δ*pyrG* strain contained a 1-bp deletion in the transcript of *pyrG* that resulted in premature translation termination. The target sequence for *pyrG* and its PAM are depicted by blue and red colors, respectively. (B) PCR verification of the *Aokap7* complemented strain C-Δ*Aokap7*. The wild-type genomic fragment of *Aokap7*, including a 1,768-bp promoter region, the entire coding sequence of *Aokap7*, and a 964-bp downstream sequence, was cloned into the pEX1 vector with the *pyrG* marker, yielding the complemented plasmid CpEX1-Aokap7. The resulting vector CpEX1-Aokap7 was introduced into Δ*Aokap7*-1-Δ*pyrG*, generating the *Aokap7* complemented strain C-Δ*Aokap7*. The complemented fragment was amplified from the genome DNA of C-Δ*Aokap7* strain using the primers C-Aokap7-F and CpEX1-R that were located in the *Aokap7* gene and the pEX1 vector, respectively. The CpEX1-Aokap7 plasmid was utilized as a template for amplifying the *Aokap7* complemented fragment to serve as a size control. (C, D) Sequence analysis of the target sites of *Aokap7* (C) and *pyrG* (D) in the complemented strain C-Δ*Aokap7*. The introduction of the wild-type genomic fragments of *Aokap7* and *pyrG* into the Δ*Aokap7*-1 mutant resulted in a heterozygous state in the C-Δ*Aokap7* strain, as evidenced by the presence of double peaks in the target sequences for *Aokap7* and *pyrG*. The target sequences and their PAM are indicated in blue and red, respectively.


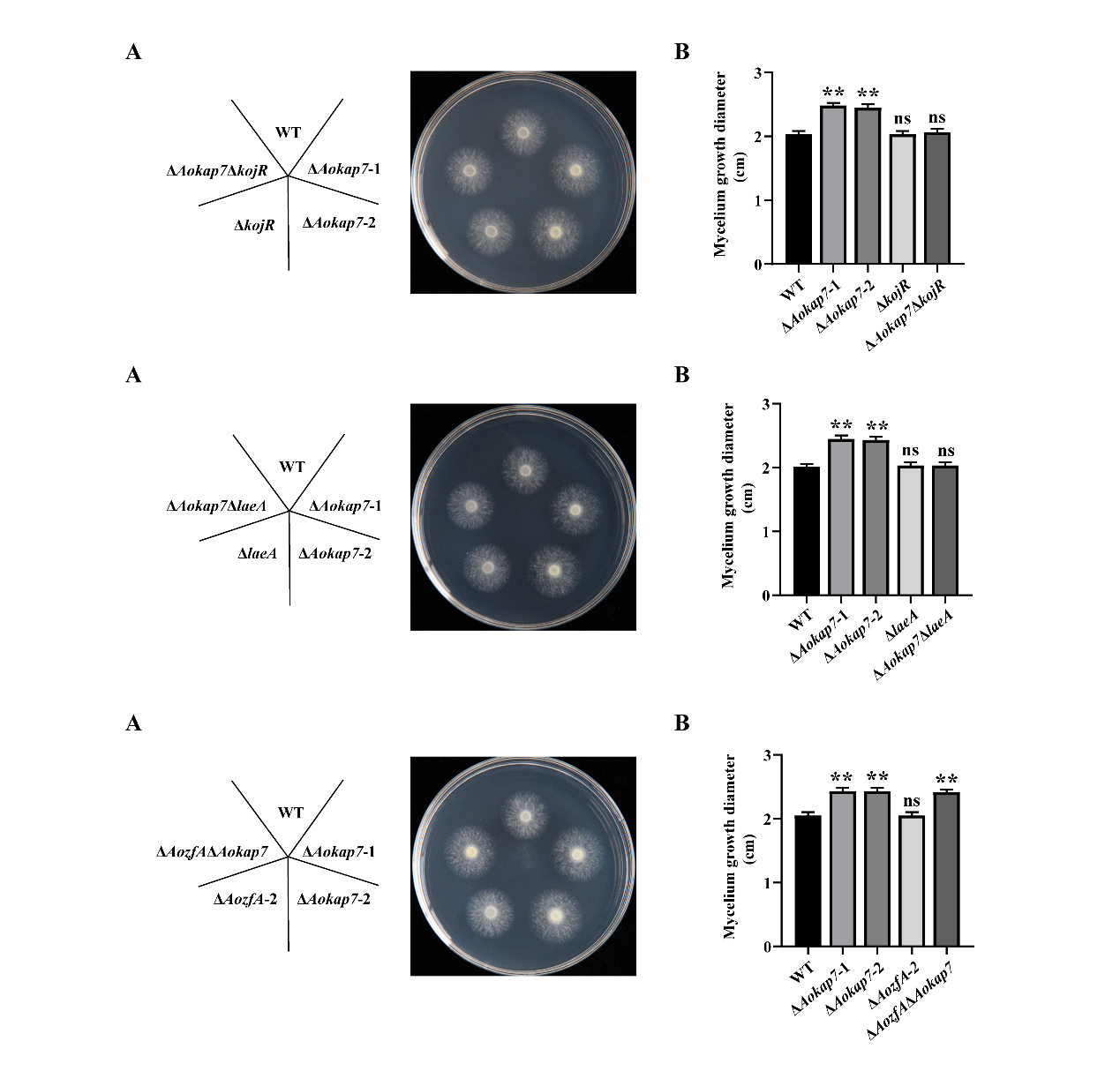


**Figure S2.** Effects of disrupting *kojR* in *Aokap7* disrupted strain on growth. (A) Phenotypes of the WT, *Aokap7* disruptants, *kojR*-disrupted mutant, double mutant of *Aokap7* and *kojR* grown on CD agar medium for 2 days. (B) Growth diameters of the WT, *Aokap7* disruptants, *kojR*-disrupted mutant, double mutant of *Aokap7* and *kojR* in panel A. Statistical significance denoted by ***p* < 0.01 when compared to the WT strain; "ns" indicates no significant difference.


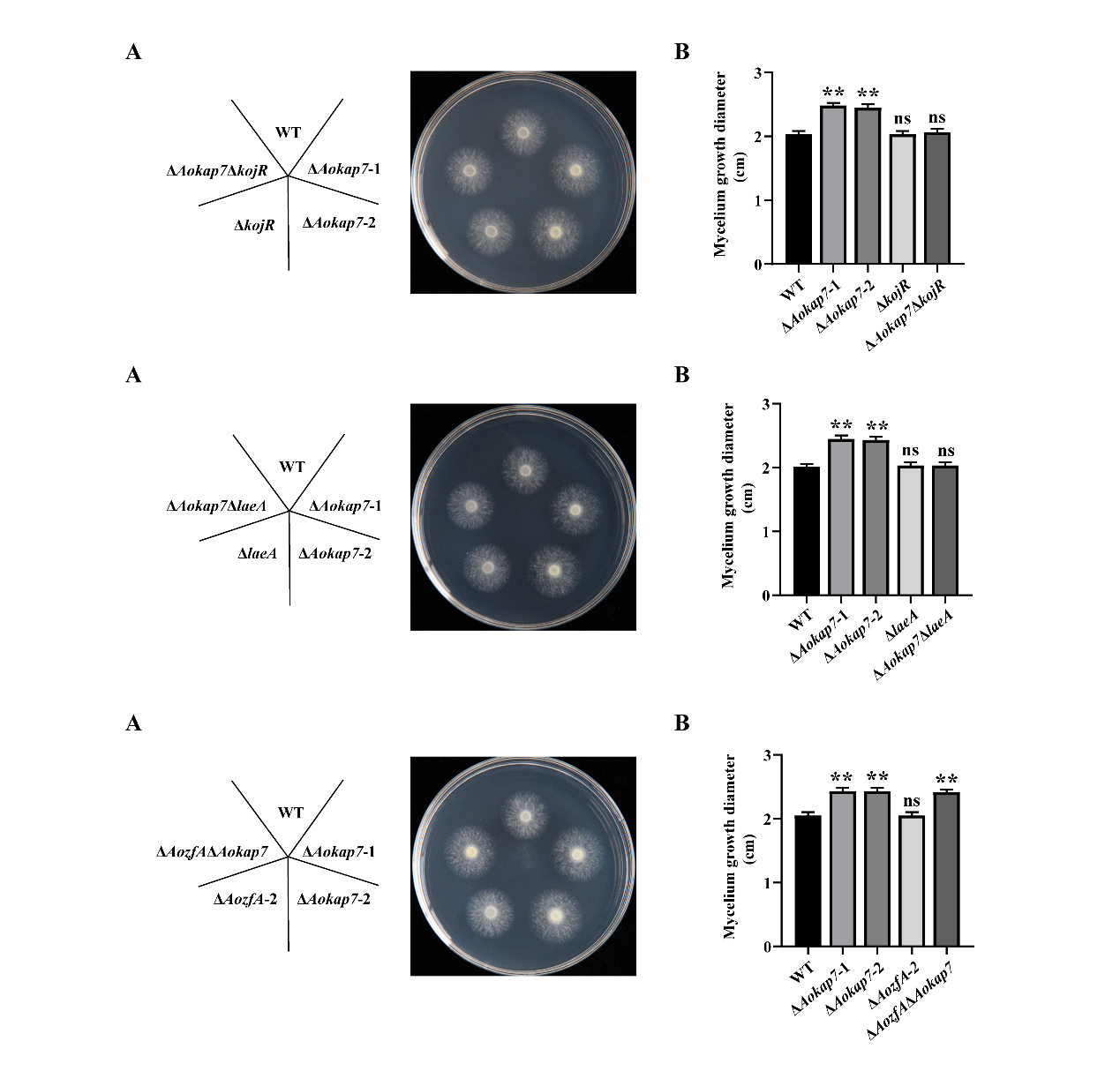


**Figure S3.** Effects of *laeA* disruption in *Aokap7* disrupted strain background on growth. (A) Growth profile of the WT, *Aokap7* disruptants, *laeA*-disrupted mutant, double mutant of *Aokap7* and *laeA* incubated on CD agar medium for 2 days. (B) Growth diameters of the WT, *Aokap7* disruptants, *laeA*-disrupted mutant, double mutant of *Aokap7* and *laeA* in panel A. Statistical significance denoted by ***p* < 0.01 when compared to the WT strain; "ns" indicates no significant difference.


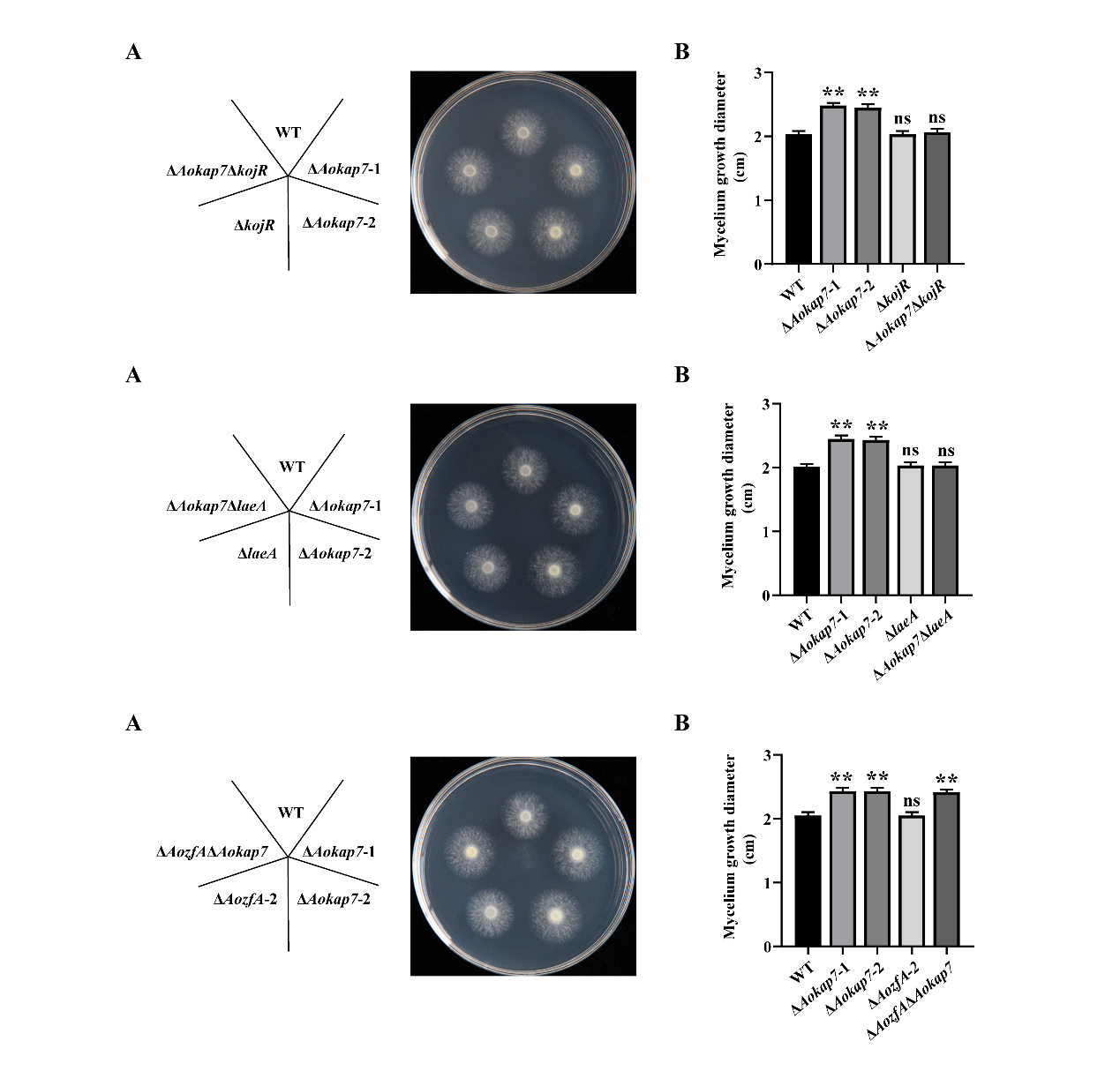


**Figure S4.** Effects of *AozfA* disruption in *Aokap7*-disrupted strain background on growth. (A) Growth of the WT, *Aokap7* disruptants, *AozfA*-disrupted mutant, double mutant of *Aokap7* and *AozfA* cultivated on CD agar medium for 2 days. (B) Growth diameters of the WT, *Aokap7* disruptants, *AozfA*-disrupted mutant, double mutant of *Aokap7* and *AozfA* in panel A. Statistical significance denoted by ***p* < 0.01 when compared to the WT strain; "ns" indicates no significant difference.


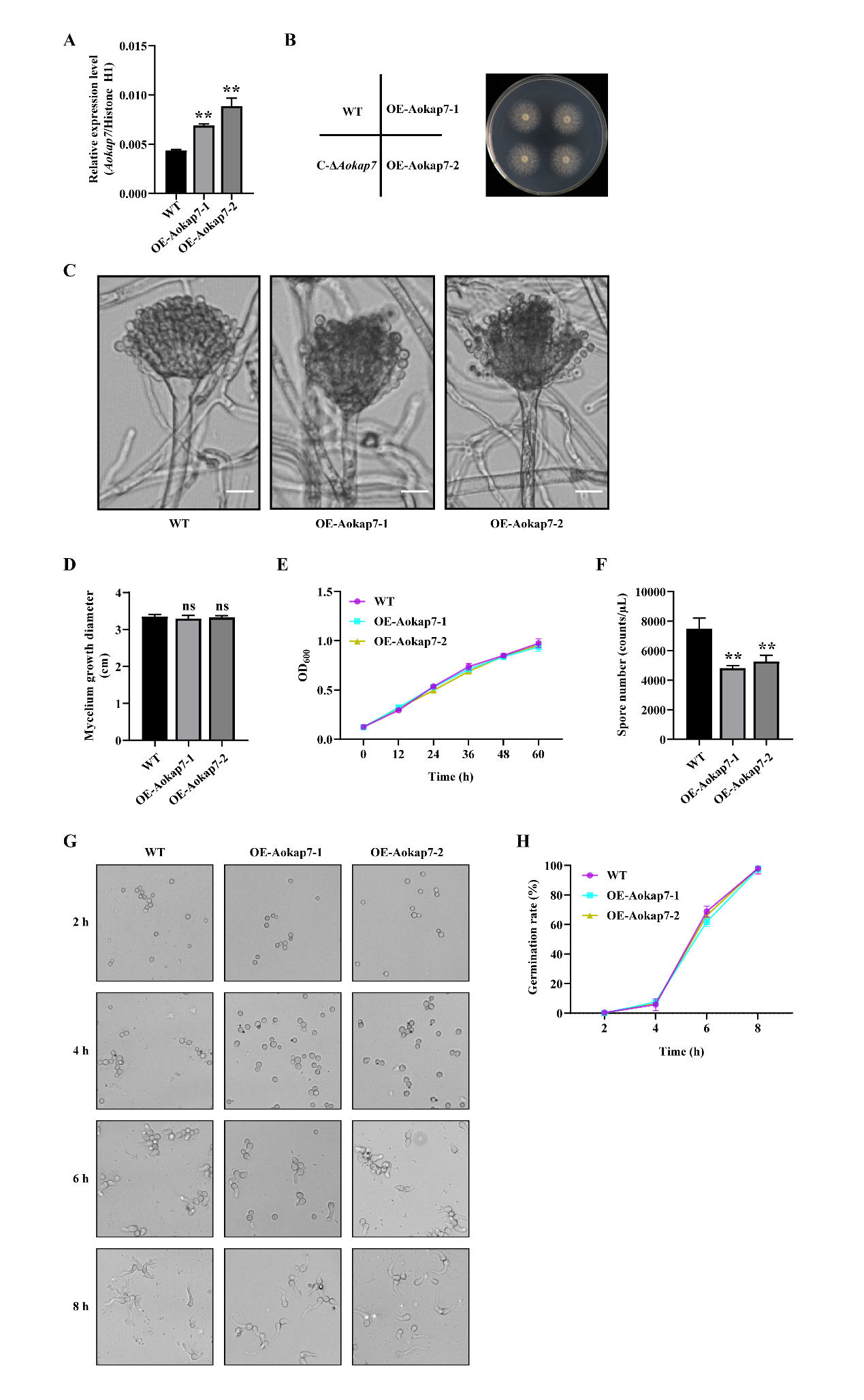


**Figure S5.** Effluences of *Aokap7* overexpression on growth. (A) The relative expression levels of *Aokap7* in the wild-type (WT) and two *Aokap7* overexpression strains cultivated in kojic acid liquid medium for 4 days. (B) Growth of the WT, OE-Aokap7-1, and OE-Aokap7-2 strains on CD agar plates for 3 days. (C) Conidiophores of the WT, OE-Aokap7-1, and OE-Aokap7-2 strains on CD agar medium for 48 h. Bar = 10 μm. (D) Growth diameters of the WT, OE-Aokap7-1, and OE-Aokap7-2 strains on CD agar medium for 3 days. (E) The growth curve of the WT, OE-Aokap7-1, and OE-Aokap7-2 strains in PDB medium.(F) Sore numbers of the WT, OE-Aokap7-1, and OE-Aokap7-2 strains on CD agar medium for 3 days. (G) The spore germination of the WT, OE-Aokap7-1, and OE-Aokap7-2 strains in PDB medium for indicated times. (H) The conidial germination rate of the WT, OE-Aokap7-1, and OE-Aokap7-2 strains in PDB medium.


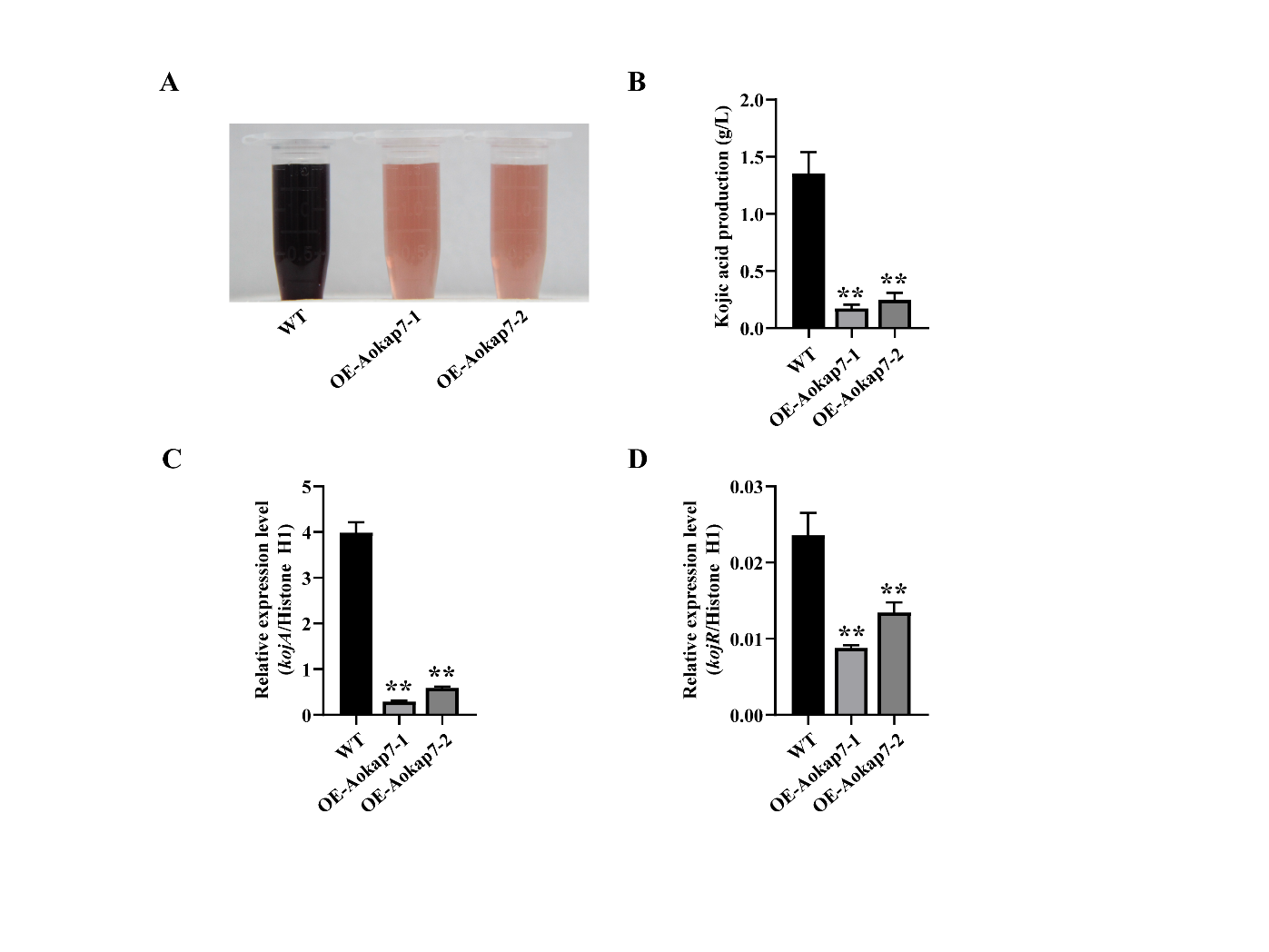


**Figure S6.** Effects of *Aokap7* overexpression on kojic acid production. (A) Colorimetric reactions of kojic acid produced by the wild-type (WT) and two *Aokap7* overexpression strains cultivated in kojic acid liquid medium for 7 days. (B) Quantification of kojic acid production in the WT and OE-Aokap7-1, and OE-Aokap7-2 strains cultivated in kojic acid liquid medium for 7 days. (C, D) The transcriptional levels of *kojA* (C) and *kojR* (D) in the WT and two *Aokap7* overexpression strains cultivated in kojic acid liquid medium for 4 days. Statistical significance denoted by ** *p* < 0.01 represents significant differences between the WT and mutant strains.


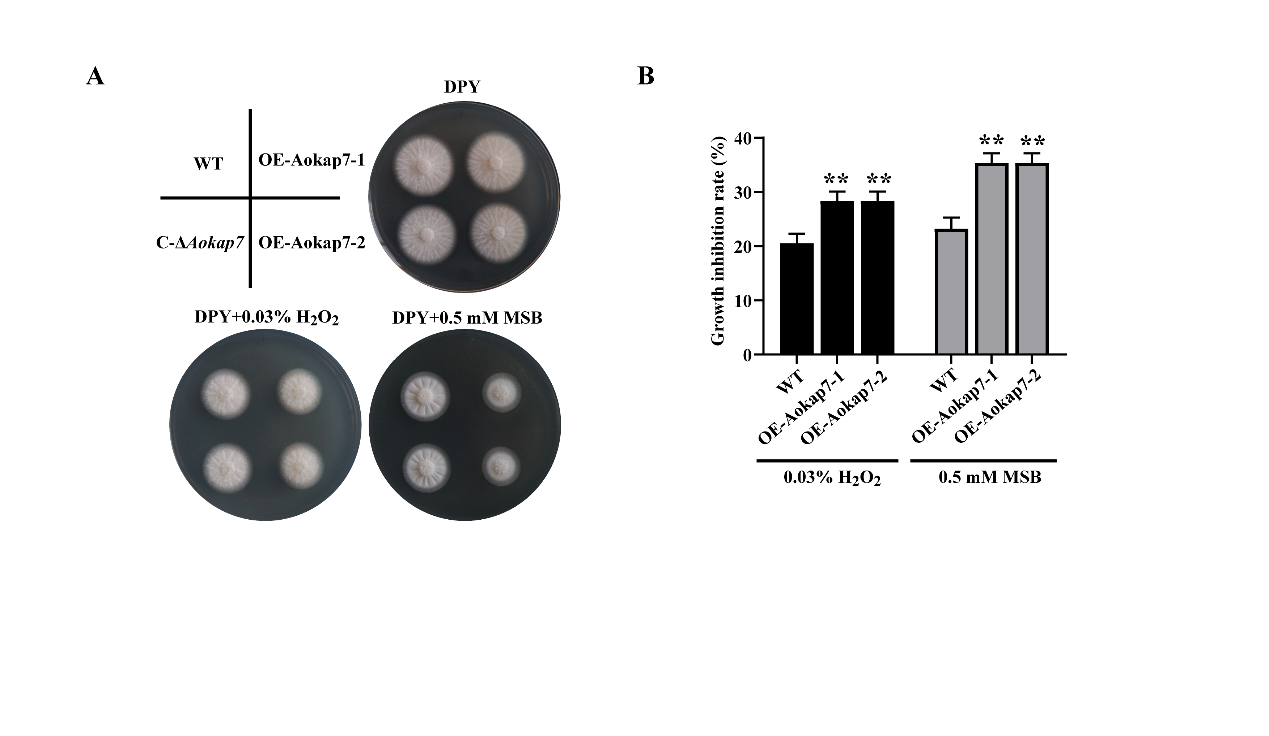


**Figure S7.** Overexpressing *Aokap7* increases sensitivity of *A. oryzae* to oxidative stress. (A) Growth of the WT, OE-Aokap7-1, OE-Aokap7-2, and C-Δ*Aokap7* strains cultured on DPY agar plates supplemented with 0.03% H_2_O_2_, or 0.5 mM MSB for 2 days. (B) The growth inhibition rate of the WT, OE-Aokap7-1, OE-Aokap7-2, and C-Δ*Aokap7* strains in panel A.

**Table S1.** Primers used in this study

| Primer name | Primer sequence (5’-3’) | Description |
| --- | --- | --- |
| PU6-Aokap7-R | TCCAGGGTCGTCGAGATATTACTTGTTCTTCTTTACAATGATTTATTTA | *Aokap7* deletion |
| TU6-Aokap7-F | AATATCTCGACGACCCTGGAGTTTTAGAGCTAGAAATAGCAAGTTAAA |  |
| PU6-F | CGACTCTAGAGGATCCCCGGGTAATGCCGGCTCATTCAAA |  |
| TU6-R | AATTCGAGCTCGGTACCCGGGAGCAGCTCTATATCACGTGACG |  |
| PU6-AoGPX1-R | TTAATCTACTTCGTCGCGCAACTTGTTCTTCTTTACAATGATTTATTTA | *AoGPX1* deletion |
| TU6-AoGPX1-F | TGCGCGACGAAGTAGATTAAGTTTTAGAGCTAGAAATAGCAAGTTAAA |  |
| CpEX1-Aokap7-F | AGCCTAGCCAACTAGTAGAACCCGACCGTTGCGTAT | Construction of the |
| CpEX1-Aokap7-R | GGCCAGTGCCAAGCTTGAGATGGAGATGGTGAGGGAGG | complemented strain |
| pEX2B-kojR-F | CGTGCCCGTGCTTAAGATGTCGTTGAATACCGACGATT | *kojR* |
| pEX2B-kojR-R | AACGTTAAGTGGATCCTTATCTATATCTCTGACCACCTGCTT | overexpression |
| pEX2B-laeA-F | CGTGCCCGTGCTTAAGATGTTTGGAAACGGCCAGACT | *laeA* |
| pEX2B-laeA-R | AACGTTAAGTGGATCCTCAGTTCGCAGGTTTCCGTG | overexpression |
| pHis2-Y4 | TGTAAAACGACGGCCAGTGAATTGTAATACGACTCACTATAGGGCGAATTCCCNNNNNNNGGGGAGCTCACGCGTTCGCGAATCGATCCGCGGTCTAGAAATTCCTGGCATTATCACATAATG | Construction of a random insertion prey library |
| pHis2-S-R | CATTATGTGATAATGCCAGG |  |
| AD-Aokap7-F | GGAGGCCAGTGAATTCATGCTGAATGATCCCACCGAC | Construct the |
| AD-Aokap7-R | CACCCGGGTGGAATTCTCAGGTCTTTCGAATGTCAGGATC | pGADT7-Aokap7 vector |
| pHis2-F | CGAAAGGGGGATGTGCTGCA | Detect DNA |
| pHis2-R | TGTGGCCTGTTCTGCTACTGC | insertion sequences |
| pHis2-3xMotif1-F | AGGGCGAATTCCCGGGCGGAGCGGCGGAGCGGCGGAGCGGCCCGGGGAGCTCACGC | Yeast one-hybrid assay |
| pHis2-3xMotif1-R | GCGTGAGCTCCCCGGGCCGCTCCGCCGCTCCGCCGCTCCGCCCGGGAATTCGCCCT |  |
| pHis2-3xMotif2-F | AGGGCGAATTCCCGGGCCCTCACCCCTCACCCCTCACCCCGGGGAGCTCACGC |  |
| pHis2-3xMotif2-R | GCGTGAGCTCCCCGGGGTGAGGGGTGAGGGGTGAGGGCCCGGGAATTCGCCCT |  |
| pHis2-pAoGPX1-F | AGGGCGAATTCCCGGGATGGCTTTCCATTCACTATTGCAT |  |
| pHis2-pAoGPX1-R | GCGTGAGCTCCCCGGGGGTGTGCGTATGAAATGCGC |  |
| pGEX-Aokap7-ZF-F | TGGATCCCCGGAATTCATGCGCCAACGCACCAGC | Expression of |
| pGEX-Aokap7-ZF-R | GTCGACCCGGGAATTCTCATTCCCTCTTTGACCCCACATA | GST-AoKap7_zf_ protein |
| AoGPX1-probe-F | ATGATGATGACATGCCGGCTCGGCGCCTTCCTCCTCCG | EMSA assay |
| AoGPX1-probe-R | CGGAGGAGGAAGGCGCCGAGCCGGCATGTCATCATCAT |  |
| rAokap7-F | ACGAAGCTGCAGAAGCACAAGC | qPCR for *Aokap7* |
| rAokap7-R | AAGTATCGCGCCCAACCAAGTG |  |
| rkojA-F | ACACAAACGAGCCCCTTCAG | qPCR for *kojA* |
| rkojA-R | CCTCGTGACGGTCGAATGA |  |
| rkojR-F | CAACTCAGGCACCGCTTTC | qPCR for *kojR* |
| rkojR-R | TCCAGCTAAACCCGTACACCT |  |
| rlaeA-F | ACCCAGACGCTTTCGTTGTTGG | qPCR for *laeA* |
| rlaeA-R | TTGGGTGGTTTGAGGGCTGTATG |  |
| rHistone-F | GACAACATCCAGGGTATCACTAAGC | qPCR for |
| rHistone-R | GGTCTCCTCGTAGATCATGGCA | histone H1 gene |
| pEX2B-F | TGGAGGATAGCAACCGACAACA | Detection of |
| pEX2B-R | CCGGAGCTGACATCGACAC | overexpression strains |
| C-Aokap7-F | TAAACGAGGGCGCTTTGTGGAC | Detection of the |
| CpEX1-R | GATGTGCTGCAAGGCGATTAAGTT | complemented strain |
